# Prevalence of incidental intracranial findings on magnetic resonance imaging: a systematic review and meta-analysis

**DOI:** 10.1007/s00701-022-05225-7

**Published:** 2022-05-08

**Authors:** Divya Elizabeth Sunny, Michael Amoo, Maryam Al Breiki, Elite Dong Wen Teng, Jack Henry, Mohsen Javadpour

**Affiliations:** 1 National Neurosurgical Centre, Beaumont Hospital, Dublin 9, Ireland; 2 School of Medicine, University College Dublin, Dublin, Ireland; 3 University of Medical and Health Sciences, Royal College of Surgeons Ireland, Dublin, Ireland; 4 Department of Academic Neurology, Trinity College Dublin, Dublin, Ireland

**Keywords:** Incidental finding, MRI, Magnetic resonance imaging, Incidentaloma, Aneurysm

## Abstract

**Background:**

As the volume and fidelity of magnetic resonance imaging (MRI) of the brain increase, observation of incidental findings may also increase. We performed a systematic review and meta-analysis to determine the prevalence of various incidental findings.

**Methods:**

PubMed/MEDLINE, EMBASE and SCOPUS were searched from inception to May 24, 2021. We identified 6536 citations and included 35 reports of 34 studies, comprising 40,777 participants. A meta-analysis of proportions was performed, and age-stratified estimates for each finding were derived from age-adjusted non-linear models.

**Results:**

Vascular abnormalities were observed in 423/35,706 participants (9.1/1000 scans, 95%CI 5.2–14.2), ranging from 2/1000 scans (95%CI 0–7) in 1-year-olds to 16/1000 scans (95%CI 1–43) in 80-year-olds. Of these, 204/34,306 were aneurysms (3.1/1000 scans, 95%CI 1–6.3), which ranged from 0/1000 scans (95%CI 0–5) at 1 year of age to 6/1000 scans (95%CI 3–9) at 60 years. Neoplastic abnormalities were observed in 456/39,040 participants (11.9/1000 scans, 95%CI 7.5–17.2), ranging from 0.2/1000 scans (95%CI 0–10) in 1-year-olds to 34/1000 scans (95%CI 12–66) in 80-year-olds. Meningiomas were the most common, in 246/38,076 participants (5.3/1000 scans, 95%CI 2.3–9.5), ranging from 0/1000 scans (95%CI 0–2) in 1-year-olds to 17/1000 scans (95%CI 4–37) in 80-year-olds. Chiari malformations were observed in 109/27,408 participants (3.7/1000 scans, 95%CI 1.8–6.3), pineal cysts in 1176/32,170 (9/1000 scans, 95%CI 1.8–21.4) and arachnoid cysts in 414/36,367 (8.5/1000 scans, 95%CI 5.8–11.8).

**Conclusion:**

Incidental findings are common on brain MRI and may result in substantial resource expenditure and patient anxiety but are often of little clinical significance.

**Supplementary Information:**

The online version contains supplementary material available at 10.1007/s00701-022-05225-7.

## Introduction

Over the past three decades, rapid technological advances have led to increased access and application of magnetic brain imaging (MRI) and computed tomography (CT) in clinical practice and research. As a consequence of improved image resolution, and a rapid rise in demand, detection of incidental findings have increased in both clinical and research context [9, 56]. Intracranial incidental findings are unintended asymptomatic abnormalities diagnosed such as brain neoplasms, aneurysms and vascular malformations [72]. Their clinical significance ranges from normal anatomical variants to pathologies that may require urgent medical or surgical interventions [49].

The prevalence of incidental findings was reported to be 18% in the first large-scale study in 1999, performed on 1000 asymptomatic volunteers (age, 3–83 years) [41]. Previous meta-analyses have reported the prevalence of incidental findings on high-resolution MRI to be 2.7% in adults and 16.4% in children [16]. Although there are some guidelines in place for managing these incidentalomas, clinicians have expressed ambivalence about the ideal management [20, 30]. When participants in the study are healthy volunteers, incidental findings can pose various practical and ethical concerns [31, 34]. The detection of these findings is potentially detrimental, as treatments are often not benign, with potentially harmful consequences [55].

A systematic review was designed to investigate estimates of the prevalence of incidental findings on brain MRI, with or without intravenous contrast, performed for clinical, commercial or research purposes in the general population. We also explored the demographic characteristics, imaging parameters and their influence on the findings. Relatively few studies have explored incidental findings on MRI in a diverse demographic including children and adults. In this review, we explore the nature, incidence and implications of intracranial incidental findings across various imaging modalities to inform patient counselling and further investigation.

## Methods

A systematic review and meta-analysis was performed according to the PRISMA guidelines [59] to determine the rate of incidental findings on brain MRI scans.

### Inclusion criteria

Studies reporting the prevalence of incidental findings on MR imaging were eligible for inclusion. Studies with significant confounding populations were excluded, such as in patients with neurosurgical referral, evidence of focal neurologic deficit or neuropsychiatric disorder. Given the well-documented associations between many incidental findings and age/gender, we excluded studies not reporting the proportions of males and females scanned and their mean/median age. Studies with scanning indications unlikely to be confounding were included, such as patients referred for assessment of headache or head trauma. In these studies, only definite incidental findings were included in our analysis. Studies which involved only healthy volunteers were examined separately as a sensitivity analysis to test the effect of this criterion, by examining the effect of including studies comprising patients with a clinical indication for brain MRI. Healthy volunteers were defined as patients with no overt neurological complaint being investigated as part of research or a screening process.

### Search strategy and selection process

PubMed, Ovid MEDLINE, EMBASE and SCOPUS were searched from inception until May 24, 2021, using the strategies in Supplementary Methods I. Citations were deduplicated using fuzzy logic matching in *revtools* [78] for *R v4.0.2*. Abstracts were then independently screened by three authors (DES, EDWT, MAB) using *Rayyan QCRI* [58], with conflicts solved by discussion with a fourth author (MA or JH). Data was extracted by three authors (DES, EDWT, MAB).

### Data abstraction

We sought data on all neoplastic, vascular or other findings identified in the included studies. Lesions identified as meningiomas by the study authors or lesions with a description consistent with a meningioma such as “calcified dural-based lesion” were considered meningiomas. Pituitary lesions include any lesion considered radiographically consistent with an adenoma, which includes micro- and macroadenomas as identified by the study authors. Undifferentiated sellar or suprasellar lesions were recorded separately. Meningiomas, gliomas and pituitary adenomas were enumerated separately given their commonality. The total number of neoplasms includes the above and any other neoplastic findings identified by the study authors, reported in the “any neoplastic” category. Neoplasms not fitting the above categories were enumerated in the “other neoplastic” category.

Cerebral aneurysms were those identified as such by the study authors and included “probable” aneurysms and thrombosed aneurysms where these were reported. We additionally enumerated cavernomas separately. The vascular category includes all vascular malformations, *including* aneurysms and cavernomas, reported as “any vascular”. Vascular findings not fitting the above were reported as “other vascular”. These included arteriovenous malformations (AVMs) and dural arteriovenous fistulas (dAVFs). Developmental venous anomalies/venous angiomas were excluded. Stenosis of a major vessel was included. In addition to vascular and neoplastic findings, we quantified the number of pineal cysts, arachnoid cysts, and Chiari malformations identified in the included studies.

Morris et al. [55] additionally found that white matter hyperintensities were the most common finding. We did not assess these because we found that the threshold for their reporting appeared to vary and was often poorly described. White matter hyperintensities exist on a spectrum from a clinically insignificant finding to pathologic white matter disease [24], but reporting thresholds are poorly standardised. This issue is exemplified in the findings of Wang et al. [76], who reported the prevalence of hyperintensities stratified into grades in 579 patients. At the lowest threshold, white matter intensities were observed in 566/579 patients (97.8%) [76]. Thus, we did not pool these findings.

### Risk of bias assessment

We assessed risk of bias within the included studies by adapting the tool proposed by Hoy et al. [32] for prevalence studies. We assessed the risk of bias in 4 domains relating to external validity and 4 domains relating to internal validity, as shown in Table 1. We assessed between-study bias using funnel plots. Conventional funnel plots using the standard error as a measure of precision may produce false-positive identification of publication bias, and thus, we generated funnel plots of log odds against sample size as suggested by Hunter et al. [33].

**Table 1:** Risk of bias classification by which studies were assessed, adopted directly from Hoy et al. [32] overall judgements are shown in Table 2

Domain	Question
*External validity*	
D1	Was the target population a close representation of the national population?
D2	Was the sampling frame a true or close representation of the target population?
D3	Was some form of random selection used to select the sample, or was a census undertaken?
D4	Was the likelihood of response bias minimal?
*Internal validity*	
D5	Were data collected directly from subjects?
D6	Was an acceptable case definition used in the study?
D7	Was the study instrument that measured the parameter of interest shown to have validity and reliability?
D8	Was the same mode of data collection used for all subjects?

**Table 2:** Characteristics of the included studies. Domain-level risk of bias findings is shown in Fig. 2

Study	*N*	Location	Age	Population	Design	Magnet	Sequences	Contrast	Assessor	Risk of bias
Serag and Ragab, 2020 [68]	753	Egypt	49.8 ± 18.7	Healthy volunteers	R-NRS	1.5 T	T1W1, T2W1, FLAIR	Y	Radiologist	High
Wang et al., 2021 [76]	579	China	67.6 ± 7.6	Healthy volunteers	P-NRS	3 T	T1W1, T2W1, FLAIR	N	Radiologist	Low
Hanna et al., 2020 [28]	125	USA	43.9 ± 15.2	Healthy volunteers	P-NRS	3 T	T1W1	N	Neuroradiologist	High
Vázquez-Justes et al., 2020 [71]	514	Spain	57	Type 2 diabetics	R-NRS	1.5 T	T1W1, T2W1, FLAIR	Y	Neuroradiologist	High
Keuss et al., 2019 [42]	471	UK	70.7	Healthy volunteers	P-NRS	3 T	T1W1 T2W1, FLAIR	N	Neuroradiologist	Low
Glasmacher et al., 2020 [22]	514	UK	60	Early-onset cognitive disorders	R-NRS	1.5 T	T1W, T2W, DWI, FLAIR	NR	NR	High
Li et al., 2019 [48]	562	China	59.3 ± 2.8	Healthy volunteers	R-NRS	3 T	T1, T2, T2-GRE, FLAIR, PDW1, PW1, DTI, TOF 3D angio	N	Neurologist	Low
Bos et al., 2016 [9]^a^	5800	Netherlands	64.9 ± 10.9	Healthy volunteers	P-NRS	1.5 T	T1W, T2W	N	Researchers w/medical degrees or training in neuropsychology	Low
Vernooij et al., 2007 [72]^a^	2000	Netherlands	63.3	Healthy volunteers	P-NRS	1.5 T	T1W1, T2W1, T2W-GRE, FLAIR	N	Neuroradiologist	Low
Li et al., 2021 [49]	11,679	USA	9.9 ± 0.62	Healthy volunteers	R-NRS	3 T	T1W1, T2W1	N	Neuroradiologist	Low
Weber and Knopf, 2006 [77]	2536	Germany	20.5	Healthy volunteers		1 T	T1W1, T2W1, T2W-GRE, FLAIR	Y	Radiologist	Low
Cohrs et al., 2018 [15]	569	Germany	9.5 ± 4.4	Mild TBI	R-NRS	1.5 T, 3 T	DW1, T2W, FLAIR	Y	Neuroradiologist	High
Alturkustani et al., 2020 [3]	275	USA	38 (IQR 30–52)	Headache	P-NRS	3 T	T1 spin-echo, T2 spin-echo, FLAIR	Y	Neuroradiologist	High
Yilmaz et al., 2014 [81]	449	Turkey	11.2 ± 3.2	Headache	P-NRS	1.5 T	T1W, T2W, FLAIR	Y	Radiologist	Low
Kim et al., 2002 [43]	225	USA	11.2	Healthy volunteers	R-NRS	NR	T1 spin-echo, T2 spin-echo	N	Neuroradiologist	High
Katzman et al., 1999 [41]	1000	USA	30.6	Healthy volunteers	R-NRS	NR	T1W, T2W	N	Radiologist	High
Onizuka et al., 2001 [57]	4000	Japan	56	Healthy volunteers	P-NRS	1 T	FLAIR	Y	NR	High
Koncz et al., 2018 [44]	400	Australia	70.4	Healthy twins	P-NRS	1.5 T	3D T1W, T2W, FLAIR	N	Neuropsychiatrist	High
Lee 2008 [64]	2164	Taiwan	51.8 ± 10.6	Healthy volunteers	P-NRS	1.5 T	NR	NR	NR	High
Brugulat-Serrat et al., 2017 [12]	575	Spain	58.2 (males) 57.5 (females)	Healthy volunteers w/FHx of Alzheimer’s	R-NRS	3 T	T1W, T2W, FLAIR, fast spin-echo, gradient-recalled echo	N	Neuroradiologist	Low
Hoggard et al., 2009 [31]	525	UK	35	Healthy volunteers	P-NRS	1.5 T, 3 T	T1W, T2W	N	Neuroradiologist	High
Boutet et al., 2017 [11]	503	France	75.3 ± 0.9	Healthy volunteers	P-NRS	1.5 T	T1W, T2W, FLAIR	N	Neuroradiologist	Low
Haberg et al., 2016 [26]	1006	Norway	59.2 ± 4.2	Healthy volunteers	P-NRS	1.5 T	T1W, ADNI, T2W, FLAIR	Y	Neuroradiologist	Low
Kaiser et al., 2015 [40]	114	USA	8.3	Healthy volunteers	R-NRS	3 T	T1W, T2W, FLAIR	N	Neuroradiologist	High
Cieszanowski et al., 2014 [14]	666	Poland	46.4 (20–77)	Healthy volunteers	R-NRS	1.5 T	T1W, T2W, STIR, FLAIR, GRE	Y	Radiologist	High
Gur et al., 2013 [25]	1400	USA	No findings: 14.7 ± 3.6 Incidental finding: 14.9 ± 3.9	Healthy volunteers	P-NRS	3 T	T1W, GRE	N	Neuroradiologist	Low
Mar et al., 2013 [52]	926	USA	12.4	Headache	R-NRS	1.5 T, 3 T	T1W, T2W, FLAIR	N	Neuroradiologist	High
Sandeman et al., 2013 [65]	700	UK	72.5	Healthy volunteers	P-NRS	1.5 T	T2W, FLAIR	Y	Neuroradiologist	Low
Potchen et al., 2013 [60]	96	USA	11.9 ± 1.5	Healthy volunteers	R-NRS	0.35 T	T1W, T2W, DWI	N	Radiologist	Low
Reneman et al., 2012 [62]	203	Netherlands	21.9 ± 3.2	Healthy volunteers	R-NRS	1.5 T, 3 T	3D T1W, T2W	Y	Head/neck radiologist or neuroradiologist	Low
Hartwigsen et al., 2010 [29]	206	Germany	25.7 ± 5.7	Healthy volunteers	P-NRS	3 T	NR	NR	Neuroradiologist	High
Lubman et al., 2002 [51]	98	Australia	27	Healthy volunteers	P-NRS	1.5 T	NR	NR	Neuroradiologist	High
Illes et al., 2004 [35]	151	USA	47.1	Healthy volunteers	R-NRS	NR	NR	NR	Neuroradiologist	High
Tsushima et al., 2005 [70]	1113	Japan	52.6 ± 8.5	Healthy volunteers	R-NRS	1 T	T1W, T2W, TOF-MRA	N	Neuroradiologist	Low
Alphs et al., 2006 [2]	656	USA	61	Lead-exposed	P-NRS	NR	T1W, T2W	NR	NR	High

^a^Reports of the same study. Data were amalgamated to maximise detail

*R-NRS* retrospective non-randomised study, *P-NRS* prospective non-randomised study, *NR* not reported, *TBI* traumatic brain injury

### Statistical analysis

All statistical analysis was performed using *R v4.0.2* [61]. As the primary outcome, we report the age-stratified prevalence (findings per 1000 scans) of the various categories of findings described above. We report crude prevalence as the secondary outcome but consider these estimates of limited value because of the extreme variation in incidences across ages. Crude estimates were derived from random effects meta-analyses of proportions with inverse variance weighting. We did not report the total prevalence of all incidental findings as an aggregate, because studies frequently described the total without fully describing the findings that contribute to it, with varying thresholds for inclusion and consequently incomparable data. We fitted mixed effects restricted cubic spline non-linear mixed effects meta-regression models, with the restricted maximum likelihood (REML) estimator using *metafor* [73]. Age-stratified estimates were then derived as fitted estimates for each age point. Fitted estimates were derived for ages 1, 2, 5 and 10 years and each decade thereafter. The continuous relationship between age and effect size was also depicted by graphing age versus the fitted spline estimates.

We also fitted linear models and reported the regression coefficient (*β*) and its 95% confidence interval (95%CI). The summary measure was the back-transformed Freeman-Tukey double arcsine-transformed proportion [50], which was chosen to stabilise variance given that findings are rare. Heterogeneity was quantified by *τ*^2^ and its impact by *I*^2^ and was derived from the random effects unadjusted crude estimates.

### Additional analyses

To assess the impact of including studies in patients with potentially neurological complaints or indications for scanning, a sensitivity analysis was performed wherein estimates were derived as described above from studies recruiting exclusively healthy volunteers/controls. As further sensitivity analyses, we also repeated the analysis using conventional weighted least squares linear regression models, both univariable models and multivariable models including both age and gender. For multivariable models, we computed fitted estimates under the assumption of an equal number of male and female participants. We also examined the effect of publication year and the use of intravenous contrast using meta-regression models, which were additionally adjusted for age. Proportions discussed in relation to these models relate to the median age in the analysis.

### Assessment of certainty

We assessed our certainty in the included findings using the GRADE framework, which downgrades certainty qualitatively based on a number of factors including the statistical effect size, its precision, the presence of heterogeneity and within-study bias.

## Results

**Fig. 1:**
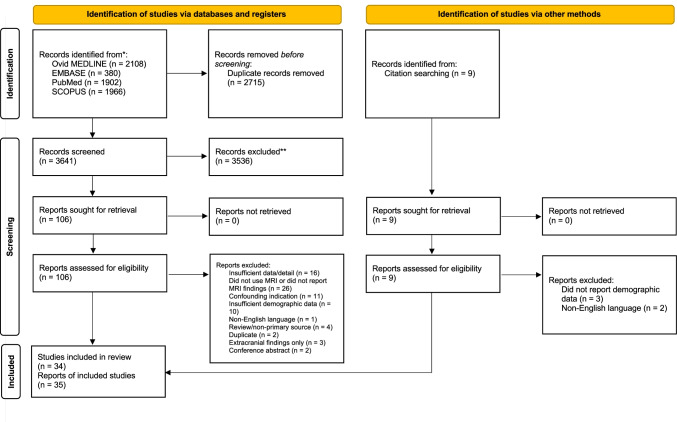
PRISMA flowchart detailing article screening and selection

A total of 6356 citations were identified, of which 106 full texts were assessed and 35 reports of 34 studies were included (Fig. 1), comprising 40,777 participants. Some studies which may appear to meet the inclusion criteria were excluded because they were published in a non-English language [5, 75] or did not report sufficient demographic detail [27, 39, 45, 46, 67, 69, 83]. Characteristics of the included studies are provided in Table 2.

### Characteristics of the included studies

**Fig. 2:**
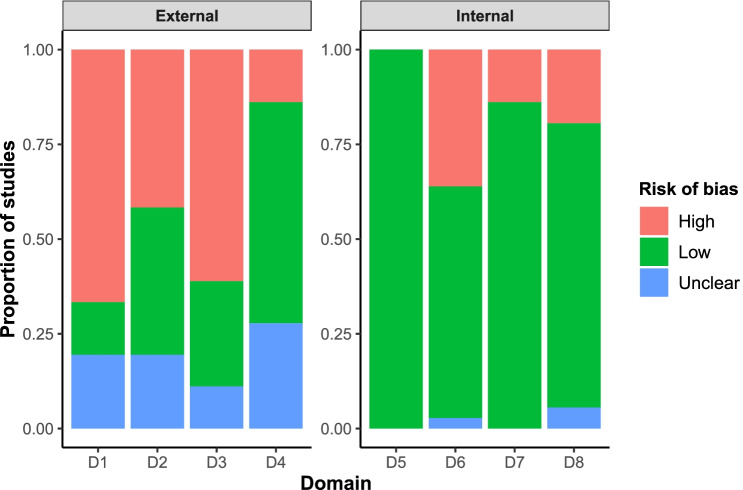
Risk of bias in each domain in the included studies

Risk of bias in each domain is summarised in Fig. 2. Studies were typically at high risk of bias in domains relating to external validity, as samples were often convenience samples or based upon self-referral. Bias relating to internal validity was generally low, because scans were by nature directly sampled from patients and MRI is sensitive and specific for the detection of intracranial abnormalities. Findings for each study are shown in Supplementary Figure I.

**Fig. 3:**
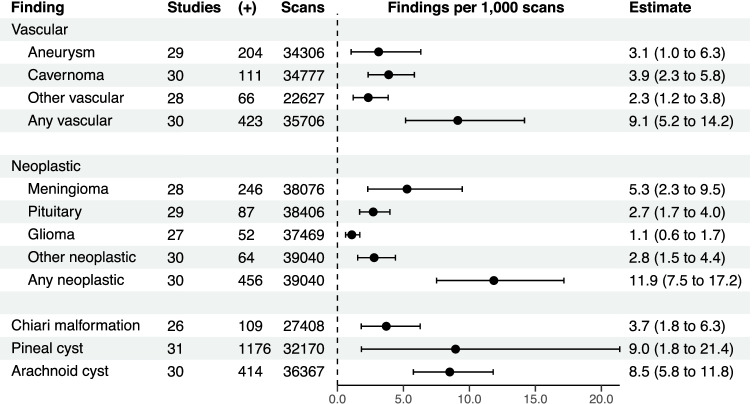
Crude estimates of the number of findings per 1000 scans in each category. ( +), number of positive scans

**Fig. 4:**
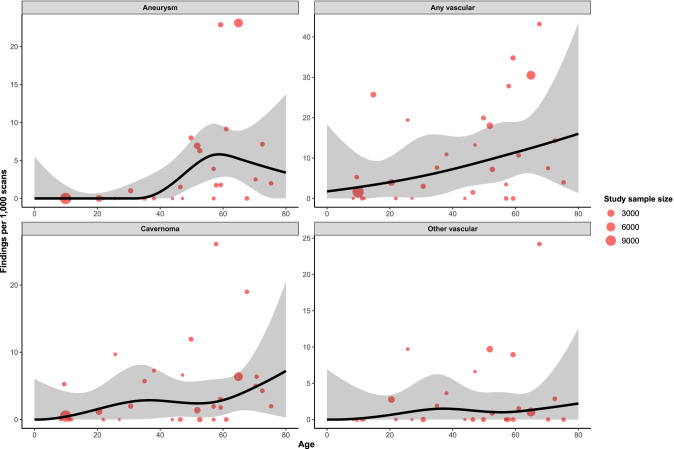
Relationship between proportion of each vascular finding and age, derived from restricted cubic spline meta-regression models. Red dots show the findings of individual studies, with the size of the point relative to study sample size. Black lines are fitted estimates, while the shaded area is the 95% confidence interval of the fitted estimate

**Fig. 5:**
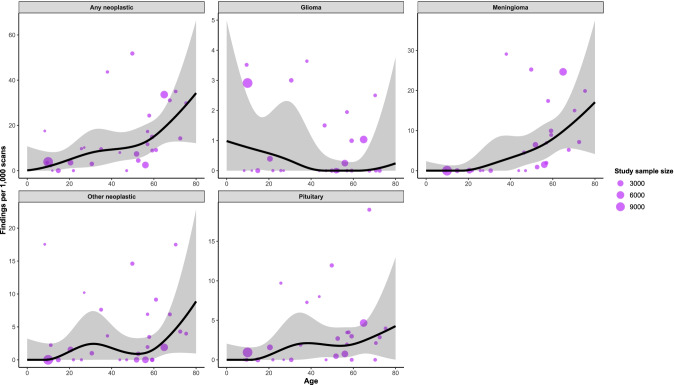
Relationship between proportion of each neoplastic finding and age, derived from restricted cubic spline meta-regression models. Purple dots show the findings of individual studies, with the size of the point relative to study sample size. Black lines are fitted estimates, while the shaded area is the 95% confidence interval of the fitted estimate

**Fig. 6:**
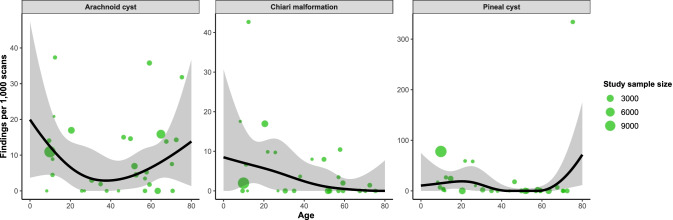
Relationship between proportion of each other finding and age, derived from restricted cubic spline meta-regression models. Green dots show the findings of individual studies, with the size of the point relative to study sample size. Black lines are fitted estimates, while the shaded area is the 95% confidence interval of the fitted estimate

Substantial heterogeneity is apparent in our analysis, which is easily appreciated by examining the vertical dispersion of effect sizes in Figs. 3, 4, 5 and 6 and forest plots in Supplementary Figure I. This may be a result of rare findings examined in relatively small sample sizes but may also be a result of differences in methodology and enrolled populations. Magnet strength and sequence differed between studies. The most frequently used strengths were 1.0 T [57, 70, 77], 1.5 T [9, 10, 14, 26, 31, 44, 51, 64, 65, 68, 71, 72, 81], and 3.0 T [3, 12, 25, 28, 29, 40, 42, 48, 49, 76], while some studies used more than one strength (1.5 T and 3 T) [15, 41, 43, 52, 62]. Imaging scans were read by senior radiologists and/or neuroradiologists and/or neurologists in a large number of the included studies [3, 12, 14, 15, 28, 40–43, 48, 49, 52, 60, 68, 71, 72, 76, 77, 81]. In one study, 2 neuropsychiatrists co-assessed the MRI scans with a neuroradiologist. Only one study [9] had no radiologist, neuroradiologist, or neurologist in the team reading the scans; their team included researchers with a doctor of medicine training in neuropsychology. T1-weighted image (T1WI) [25, 28], T2-weighted image (T2WI) and fluid-attenuated inversion recovery (FLAIR)[57] were the most commonly used MRI sequences, either alone or combined [3, 9, 10, 12, 14, 15, 26, 31, 40–42, 44, 48, 49, 52, 62, 65, 68, 71, 72, 76, 77, 81]. Other sequences that were used in a smaller number of published papers were gradient recalled echo T2WI (GRE T2WI) [12, 14, 48, 72, 77], proton density weighted imaging (PDWI) [48], perfusion weighted imaging (PWI) [48], diffusion tensor imaging (DTI) [48], time-of-flight (TOF) [48] angiography and T1/T2 spine echo (SE) [43]. Of the 35 included studies, 11 used a contrast agent [3, 14, 15, 26, 57, 62, 65, 68, 71, 77, 81], which improves the sensitivity of imaging [13].

There was also substantial variation in patient age, with studies focusing on children [15, 25, 40, 43, 49, 52, 60, 81], adults [3, 14, 28, 29, 31, 41, 62, 68, 77] or the elderly population [9, 10, 22, 26, 42, 44, 48, 57, 64, 65, 71, 72, 76]. This poses substantial analytic challenges. While meta-regression can approximate the relationship between age and prevalence, it is important to note that this approximation may be less valid in the context of rare findings. Ideally, future studies should consider pooling individual patient data to better characterise the relationship between findings and age. Most studies focused on healthy elderly [9, 10, 26, 42, 44, 48, 65, 72, 76], healthy adults [14, 29, 31, 41, 57, 62, 64, 68, 77] and healthy children [25, 40, 49, 60]. Three papers were on children who presented with headaches [43, 52, 81], while one was on adults with headaches [3]. Children with TBI [15], lead-exposed patients [2], patients with diabetes [71] and patients with early Alzheimer’s disease [12] were also the focus for some of the research done. While their inclusion may be considered confounding, excluding these studies in a sensitivity analysis did not substantially influence our results (Supplementary Figure II; Supplementary Table III). An element of selection bias is also possible in that populations were often self-selected volunteers or commercial screening populations, which may be reflective of socio-economic status or other social determinants of health.

### Findings

Crude estimates for each category are shown in Fig. 3. Age-stratified estimates of the prevalence of findings per 1000 scans are shown in Table 3. Findings comprising the “other” category are shown in Table 4.

**Table 3:** Age-stratified findings per 1000 scans. Numbers in parentheses represent the 95% confidence interval. Findings derived from univariable restricted cubic spline meta-regression models

			Age in years										
Finding	*τ*^2^	I^2^	1	5	10	20	30	40	50	60	70	80	Grade
Vascular													
*Aneurysm*	0.0036	94%	0 (0–5)	0 (0–3)	0 (0–2)	0 (0–0.7)	0 (0–2)	0.9 (0–4)	4 (1–8)	6 (3–9)	5 (1–10)	3 (0–14)	Low
*Cavernoma*	0.001	80%	0.00001 (0–6)	0.1 (0–5)	0.4 (0–4)	2 (0.003–5)	3 (0.03–8)	3 (0.3–7)	2 (0.4–6)	3 (0.7–6)	5 (1–10)	7 (0.3–20)	Low
*Other vascular*	0.0008	71%	0 (0–7)	0.02 (0–5)	0.1 (0–4)	0.6 (0–3)	1 (0–6)	1 (0–6)	1 (0––4)	1 (0–4)	2 (0–6)	2 (0–13)	Very low
*Any vascular*	0.0037	94%	2 (0–17)	2 (0–14)	3 (0–11)	4 (0.4–10)	5 (0.3–15)	7 (2–15)	9 (4–17)	11 (6–19)	14 (5–26)	16 (1–43)	Very low
Neoplastic													
*Meningioma*	0.0038	95%	0 (0–2)	0 (0–2)	0 (0–1)	0.1 (0–2)	1 (0–7)	3 (0.2–9)	5 (2–10)	8 (4–13)	12 (5–21)	17 (4–37)	Low
*Pituitary*	0.0005	69%	0 (0–2)	0 (0–2)	0 (0–2)	0.6 (0–2)	2 (0–5)	2 (0.2–5)	2 (0.3–4)	2 (0.5–4)	3 (0.6–7)	4 (0.03–13)	Low
*Glioma*	0.0001	40%	1 (0–5)	0.9 (0–4)	0.8 (0.0003–2)	0.6 (0–2)	0.3 (0–2)	0.06 (0–1)	0 (0–0.4)	0 (0–0.3)	0.05 (0–1)	0.2 (0–4)	Low
*Other neoplastic*	0.0009	81%	0 (0–3)	0 (0–3)	0.03 (0–2)	1 (0–4)	2 (0.007–7)	2 (0.02–5)	0.9 (0–3)	2 (0.2–4)	5 (1–10)	9 (1–23)	Very low
*Any neoplastic*	0.0033	94%	0.2 (0–10)	0.8 (0–9)	2 (0–8)	5 (1–11)	8 (2–18)	9 (3–18)	11 (5–18)	15 (9–22)	24 (13–37)	34 (12–66)	Very low
Chiari malformation	0.0017	85%	8 (0–29)	8 (0.3–23)	7 (1–16)	6 (1–13)	4 (0.07–12)	2 (0.002–8)	1 (0–6)	0.5 (0–3)	0.1 (0–4)	0 (0–8)	Low
Pineal cyst	0.0211	99%	11 (0–71)	13 (0–57)	15 (0.7–44)	19 (4–44)	12 (0–41)	1 (0–13)	0 (0–4)	0.6 (0–10)	22 (3–55)	72 (12–175)	Very low
Arachnoid cyst	0.0016	87%	19 (4–44)	16 (4–34)	12 (5–23)	7 (2–14)	4 (0–12)	3 (0.02–9)	4 (0.6–10)	7 (2–12)	10 (3–19)	14 (1–37)	Low

**Table 4:** Findings comprising the “other” category in each analysis

Vascular		Neoplastic	
Finding	*N*	Finding	*N*
Venous malformation	14	Lipoma	18
dAVF	4	Metastasis	3
AVM	12	DNET	2
Missing AComm	1	Vestibular schwannoma	2
Kinking of ICA	1	Osteoma	2
Significant ICA stenosis	4	Craniopharyngioma	1
Significant MCA stenosis	12	Skull base tumour	1
Significant PCA stenosis	3	Subcortical nodule	1
Significant VA stenosis	5	Trigeminal schwannoma	3
ICA occlusion	7	Cerebellar lesion	4
Major vessel stenosis	1	Ganglioglioma	1
Other*	2	Subependymoma	3
		Pineocytoma	1
		CPA tumour	1
		4^th^ ventricle tumour	2
		Intraventricular tumour	1
		Cerebral tumour	1
		Corpus callosum tumour	1
		Arachnoid/cystic neoplasm	1
		Choroid plexus neoplasm	1
		Hamartoma	1
		Epidermoid	1
		Other*	12

*Findings not further classified, for example “4 neoplasms”

*dAVF* dural arteriovenous fistula, *AVM* arteriovenous malformation, *AComm* anterior communicating artery, *ICA* internal carotid artery, *MCA* middle cerebral artery, *PCA* posterior cerebral artery, *VA* vertebral artery, *DNET* dysembryoplastic neuroepithelial tumour, *CPA* cerebellopontine angle

#### Vascular findings

Cavernomas were the most common vascular finding, observed in 111/34,777 participants (3.9/1000 scans, 95%CI 2.3–5.8), with a range of 0.00001/1000 scans (95%C 0–6) in 1-year-olds to 7/1000 scans (95%CI 0.3–20) in 80-year-olds (Fig. 4). There appeared to be a linear relationship between the proportion of vascular findings observed and increasing patient age (*β* = 0.002, *p* < 0.0001; Supplementary Table I). Substantial heterogeneity was observed in effect sizes for vascular findings (*τ*^2^ = 0.004, *I*^2^ = 94%) (Table 3), likely due to the substantial range of demographics of the included studies.

#### Neoplastic findings

Meningiomas were the most common neoplastic finding, observed in 246/38,076 participants (5.3/1000 scans, 95%CI 2.3–9.5), ranging from 0/1000 scans (95%CI 0–2) in 1-year-olds to 17/1000 scans (95%CI 4–37) in 80-year-olds (Fig. 5). There was a linear association with age (*β* = 0.002, *p* < 0.0001; Supplementary Table I). The rate of findings for aggregated neoplasms was 456/39,040 (11.9/1000 scans, 95%CI 7.5–17.2), ranging from 0.2/1000 scans (95%CI 0–10) in 1-year-olds to 34/1000 scans (95%CI 12–66) in 80-year-olds. Moderate heterogeneity was observed (*τ*^2^ = 0.003, *I*^2^ = 94%) (Table 3). The proportions of chiari malformations, pineal cysts and arachnoid cysts in relation to age are shown in Fig. 6.

### Additional analyses

Results of multivariable regressions are shown in Supplementary Table II. A sensitivity analysis limited to healthy volunteers did not appear to alter the relationships between age and findings (Supplementary Figure II) and did not substantially alter our age-stratified estimates (Supplementary Table III). Using linear models appeared to alter the apparent relationship between age and effect size in some analyses, particularly for those appearing to have bimodal relationships in non-linear models (Supplementary Figure III). However, this did not substantially alter our pooled estimates (Supplementary Table IV). Adjusting linear models for gender did not appear to have a substantial effect on the observed relationships (Supplementary Figure IV) or pooled estimates (Supplementary Table V). Funnel plots did not show evidence of publication bias (Supplementary Figure V).

**Fig. 7:**
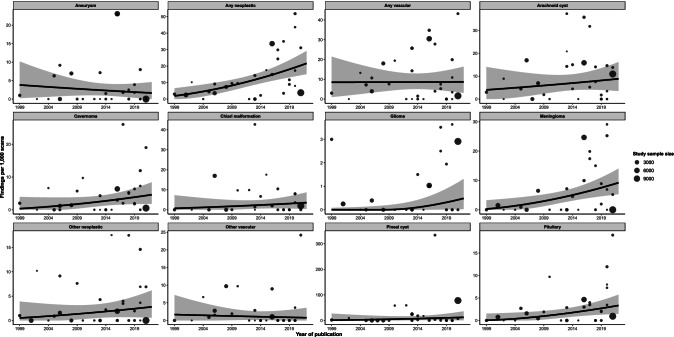
Relationship between proportion of findings and publication year, derived from multivariable meta-regression models additionally adjusted for age. Proportions on the *y*-axis relate to median age in the given analysis. Points show the findings of individual studies, with the size of the point relative to study sample size. Black lines are the fitted estimates, while the shaded area is the 95% confidence area of the fitted estimate

We found that the prevalence of neoplastic findings appeared to increase substantially in newer versus older studies, after adjustment for age (*β* = 0.004, *p* < 0.001) but not vascular findings (*β* = 0.00004, *p* = 0.98) (Fig. 7). Regression coefficients for all analyses are shown in Supplementary Table VI. The use of intravenous contrast did not appear to influence the age-adjusted proportion of neoplastic (13.2/1000 scans vs. 12.7/1000 scans, *p* = 0.92) or vascular findings (8.3/1000 scans vs. 8.8/1000 scans, *p* = 0.91) (Supplementary Table VII).

### Summary of findings

GRADE judgements of certainty are provided in Table 3 for each analysis. We had predominantly low certainty, with estimates downgraded primarily for imprecision, indirectness and within-study bias.

## Discussion

This meta-analysis identified a substantial prevalence of various incidental findings on brain MRI, which were most commonly meningiomas. Our analysis included predominantly healthy populations and thus may reasonably approximate the prevalence of these findings in the healthy population. In keeping with a previous analysis by Morris et al. [55], we identified a point prevalence of neoplastic findings of approximately 10 per 1000 scans, with a significant increase with older age. In their study [55], aneurysms were the most common vascular finding. We found a similar prevalence of aneurysms but a higher prevalence of cavernomas, which were the most common vascular finding in our study. Our analysis includes an additional 18 studies (21,218 patients) with a high prevalence of cavernomas in some large studies [9, 12, 42, 68, 76], which accounts for this discrepancy. The reason for this difference is unclear but is likely to be age-related given that we observed substantial correlation with age. This underscores the importance of interpreting crude prevalence with caution in the presence of a strong covariate. Like Morris et al. [55], we also identified a substantial correlation with age for most findings. However, we observed a bimodal relationship with age for arachnoid cysts and a decreasing incidence of Chiari malformations with age. Gliomas were insufficiently common to reliably determine any relationship with age, but an increasing incidence with age is well described in the population [80].

The clinical approach to incidental intracranial findings is uncertain. In particular, the management of common findings such as meningiomas, pituitary adenomas, cavernomas and aneurysms has been the subject of much debate. In the case of meningiomas, treatment for symptomatic presentations is clear in that maximal safe resection is typically the first-line option where feasible [23]. Conversely, the majority of asymptomatic, incidental meningiomas will not require surgical intervention, and the natural history of these lesions is uncertain [36, 37]. Studies have attempted to develop prognostic schema [36], but there are currently no well-validated tools. The management of incidental intracranial aneurysms is also subject to debate [4, 7, 17, 54, 79]. The incidence of aneurysms [74] appears to be significantly larger than that of aneurysmal subarachnoid haemorrhage (aSAH) [18]. Approximately two thirds of aneurysms in the population are < 5 mm in size [74] which, in most cases, appear to have a low risk of rupture [4, 7, 17]. Thus, even in the endovascular era, the risks of treatment may often outweigh the risk of rupture [79].

This then poses an important ethical question as to whether patients should be informed of incidental findings of questionable significance, especially when those findings are unlikely to require treatment [31]. Knowledge of these findings may cause substantial anxiety [21, 38], and thus this question is of particular importance in the context of imaging performed for research purposes in otherwise healthy individuals [31]. In addition, an increase in imaging utilisation [1] and fidelity may lead to a larger volume of incidental findings. Another consideration is a unprecedented level of access to radiology reports by patients [47, 53]. Arguably, explicit consent should be obtained with careful consideration of the implications of incidental findings, and clear thresholds at which findings are considered reportable should be determined [31]. This is particularly important when imaging studies are read by non-radiologists for research purposes [31]. In these situations, review by specialists should ideally be obtained before findings are disclosed to patients and/or further action is taken [31]. Inter-rater reliability should also be accounted for, given that disagreement may occur as to what warrants further evaluation. This is exemplified by incidental pituitary lesions—one study identified pituitary abnormalities in over 40% of patients, but with significant inter-rater variability [27]. The majority of small (≤ 1 cm) non-functioning adenomas will never enlarge [19], and thus, there is the question as to the approach to lesions with disagreement between evaluators. As such, clear guidelines and algorithms should arguably be enacted to facilitate consistent decision-making. These may also be of benefit when considering medico-legal implications[6], as decisions to forego treatment may be scrutinised in the event of preventable complications of a known, but untreated, pathology.

Incidental findings also carry economic implications [21]. It is important to note that there is no evidence of a concrete, patient-centred benefit to their identification. In a study of 5800 patients, Bos et al. [9] identified 143 meningiomas. Of these, 91 (63.6%) were referred for further assessment and only 15 (10.4%) required intervention [9]. In total, 188 of 549 findings (32%) required specialist referral and only 44 (8%) required further follow-up or intervention; one for every 131 scans performed [9]. Thus, it appears that approximately one third of incidental findings create additional workload in the form of specialist referral, but only a minority require further intervention or follow-up. This carries cost and anxiety for the patient, but may infrequently result in intervention, and thus the risk–benefit balance is uncertain. As an example, the utility of empiric screening for intracranial aneurysms has been highly debated given their commonality, the devastating outcomes of aSAH and the existence of an effective treatment [63]. However, even in a Japanese population known to be at particularly high-risk of aSAH [18], Yoshimoto et al. found that empiric screening is not cost-effective [82].

For patients, there are also implications for underwriting of personal insurance and bank loans. The presence of an intracranial finding may increase premium rates or even exclude the applicant entirely. For example, the presence of multiple cerebral aneurysms, suggesting a genetic component, or untreated aneurysms with high risk features may preclude underwriting [66]. This may seriously affect patients, especially younger individuals yet to obtain life insurance or mortgages. These consequences are often overlooked by patients and clinicians, but it is recommended they are explicitly discussed with patients undergoing imaging for screening or research purposes [8].

### Limitations

There are several limitations to this study. Firstly, we observed substantial variation in methodological components of included studies, such as magnet strengths and sequences. This was reflected in substantial heterogeneity in many analyses, which limits the certainty of our findings. Perhaps more importantly, the reporting threshold for incidental findings was not standardised in our analysis and was often undefined or unclear in the included studies. As imaging fidelity improves, the size threshold for the visual detection of lesions is becoming smaller, and thus, it is possible and even likely that this threshold varied across the studies, which may bias estimates. Given the very strong relationship between mean age in the study and the prevalence of findings identified, raw proportions for each finding may be less informative. The enrolled populations in included studies also varied substantially, which may influence baseline risk for the various findings assessed and bias our estimates. We observed substantial heterogeneity in many cases, which limits our confidence. This analysis assesses predominantly outwardly healthy individuals and thus generalisability to hospital populations, in which most incidental findings are identified in practice, may be limited.

## Conclusion

We identified a substantial prevalence of incidental findings on MRI brain in predominantly healthy volunteers. Meningiomas appear to be the most common of these, though their prevalence is highly age-dependent. The significance and optimal management of incidental findings is uncertain, and future studies should consider reporting their natural history and clinical course. Future reviews should consider obtaining individual patient data to better describe the relationship between age and prevalence.

## Supplementary Information

Below is the link to the electronic supplementary material.

Supplementary file1 (PDF 2034 KB)

## Data Availability

The datasets from which these results are generated are collated from the literature and available upon reasonable request to the corresponding author.

## Statistical code

The statistical code used to generate these results is available upon reasonable request to the corresponding author.
