# Supplementary material for: Prevalence of incidental intracranial findings on magnetic resonance imaging: a systematic review and meta-analysis
Source: Acta Neurochir (Wien). 2022 May 8;164(10):2751–65. doi: 10.1007/s00701-022-05225-7 (PMC9519720; doi:10.1007/s00701-022-05225-7)
Supplement: Supplementary file 1 — Supplementary file1 (PDF 2034 KB) [file 701_2022_5225_MOESM1_ESM.pdf]

Supplementary Information

Prevalence of incidental jntracranial findings on magnetic resonance imaging: A systematic review and meta-analysis

Divya Elizabeth Sunny[1, 2], Michael Amoo, MB, MCh, MRCS[1, 3], Maryam Al Breiki[1,2], Elite Dong Wen Teng[1,2], Jack Henry, BSc[1,2], Mohsen Javadpour, MB, BCh, FRCS(SN)[1, 3, 4]

Author Affiliations

1. National Neurosurgical Centre, Beaumont Hospital, Dublin, Ireland.
2. School of Medicine, University College Dublin, Dublin, Ireland.
3. University of Medical and Health Sciences, Royal College of Surgeons Ireland, Dublin, Ireland.
4. Department of Academic Neurology, Trinity College Dublin, Dublin, Ireland

Corresponding Author
Michael Amoo
National Neurosurgical Centre, Beaumont Hospital, Dublin 9, Ireland
E: michaelamoo@rcsi.ie

Post-publication Correspondence:
Michael Amoo
National Neurosurgical Centre, Beaumont Hospital, Dublin 9, Ireland
E: michaelamoo@rcsi.ie
Twitter: @mikeamoojr

Prof. Mohsen Javadpour
National Neurosurgical Centre, Beaumont Hospital, Dublin 9, Ireland
E: mjavadpour@rcsi.ie

Supplementary Methods I

Ovid MEDLINE – Inception to 24/05/2021 – 2,108 results

#	Query	Results from 24 May 2021
1	incidental.ti,ab.	28,619
2	exp Incidental Findings/	10,892
3	ct.ti,ab.	362,366
4	exp Magnetic Resonance Imaging/ or exp Tomography, X-Ray Computed/	847,759
5	mri.ti,ab.	258,115
6	magnetic resonance*.ti,ab.	358,887
7	computed tomography.ti,ab.	263,390
8	exp Brain/	1,238,587
9	neuroradiology.ti,ab.	2,783
10	neuro*.ti,ab.	1,890,201
11	brain.ti,ab.	1,019,915
12	head.ti,ab.	334,357
13	cranial.ti,ab.	76,967
14	cerebral.ti,ab.	362,919
15	1 or 2	34,715
16	3 or 4 or 5 or 6 or 7	1,273,989
17	8 or 9 or 10 or 11 or 12 or 13 or 14	3,326,774
18	15 and 16 and 17	2,486
19	limit 18 to humans	2,108

EMBASE – Inception to 24/05/2021 – 380 results

Search Queries

No.	Query	Results	Date
#17	(('incidental finding'/exp OR 'incidental finding') AND ('computer assisted tomography'/exp OR 'ct':ab,ti OR 'computed tomography':ab,ti OR 'neuroradiology'/exp OR 'nuclear magnetic resonance imaging'/exp OR 'magnetic resonance imaging':ab,ti OR 'mri':ab,ti) AND ('brain'/exp OR neuro*:ab,ti OR head:ab,ti OR cranial:ab,ti)) AND [embase]/lim NOT ([embase]/lim AND [medline]/lim) NOT 'conference abstract'/it	380	24 May 2021
#16	(('incidental finding'/exp OR 'incidental finding') AND ('computer assisted tomography'/exp OR 'ct':ab,ti OR 'computed tomography':ab,ti OR 'neuroradiology'/exp OR 'nuclear magnetic resonance imaging'/exp OR 'magnetic resonance imaging':ab,ti OR 'mri':ab,ti) AND ('brain'/exp OR neuro*:ab,ti OR head:ab,ti OR cranial:ab,ti)) AND [embase]/lim NOT ([embase]/lim AND [medline]/lim)	1011	24 May 2021
#15	('incidental finding'/exp OR 'incidental finding') AND ('computer assisted tomography'/exp OR 'ct':ab,ti OR 'computed tomography':ab,ti OR 'neuroradiology'/exp OR 'nuclear magnetic resonance imaging'/exp OR 'magnetic resonance imaging':ab,ti OR 'mri':ab,ti) AND ('brain'/exp OR neuro*:ab,ti OR head:ab,ti OR cranial:ab,ti)	2249	24 May 2021
#14	'brain'/exp OR neuro*:ab,ti OR head:ab,ti OR cranial:ab,ti	3758698	24 May 2021
#13	'computer assisted tomography'/exp OR 'ct':ab,ti OR 'computed tomography':ab,ti OR 'neuroradiology'/exp OR 'nuclear magnetic resonance imaging'/exp OR 'magnetic resonance imaging':ab,ti OR 'mri':ab,ti	2203543	24 May 2021
#12	cranial:ab,ti	105073	24 May 2021
#11	head:ab,ti	449588	24 May 2021
#10	neuro*:ab,ti	2534691	24 May 2021
#9	'brain'/exp	1521819	24 May 2021
#8	'mri':ab,ti	443977	24 May 2021
#7	'magnetic resonance imaging':ab,ti	292875	24 May 2021
#6	'nuclear magnetic resonance imaging'/exp	1023815	24 May 2021
#5	'neuroradiology'/exp	90889	24 May 2021
#4	'computed tomography':ab,ti	329440	24 May 2021
#3	'ct':ab,ti	610775	24 May 2021
#2	'computer assisted tomography'/exp	1175732	24 May 2021
#1	'incidental finding'/exp OR 'incidental finding'	23805	24 May 2021

PubMed – Inception to 24/05/2021 – 1,902 results

Search: **(((incidental[Title/Abstract]) OR ("incidental findings"[MeSH Major Topic])) AND (((((Brain[MeSH Terms]) OR (brain[Title/Abstract])) OR (head[Title/Abstract])) OR (cranial[Title/Abstract])) OR (neuro*[Title/Abstract]))) AND ((((MRI[Title/Abstract]) OR (magnetic resonance[Title/Abstract])) OR ("magnetic resonance imaging"[MeSH Major Topic])) OR (((computed tomgraphy[Title/Abstract]) OR (ct[Title/Abstract])) OR (computed tomography, x ray[MeSH Terms])))** Sort by: **Most Recent**
("incidental"[Title/Abstract] OR "incidental findings"[MeSH Major Topic]) AND ("brain"[MeSH Terms] OR "brain"[Title/Abstract] OR "head"[Title/Abstract] OR "cranial"[Title/Abstract] OR "neuro*"[Title/Abstract]) AND ("MRI"[Title/Abstract] OR "magnetic resonance"[Title/Abstract] OR "magnetic resonance imaging"[MeSH Major Topic] OR ("computed tomgraphy"[Title/Abstract] OR "ct"[Title/Abstract] OR "tomography, x ray computed"[MeSH Terms]))
Translations
Brain[MeSH Terms]: "brain"[MeSH Terms]
computed tomography, x ray[MeSH Terms]: "tomography, x-ray computed"[MeSH Terms]

SCOPUS – Inception to 24/05/2021 – 1,966 results

1. TITLE-ABS-KEY("incidental") **(52,804 results)**
2. TITLE-ABS-KEY("MRI") OR TITLE-ABS-KEY("magnetic resonance") OR TITLE-ABS-KEY(mr*) **(2,453,606 results)**
3. TITLE-ABS-KEY("CT") OR TITLE-ABS-KEY("computed tomography") OR TITLE-ABS-KEY("computed tomographic") **(827,684 results)**
4. TITLE-ABS-KEY("head") OR TITLE-ABS-KEY("brain") OR TITLE-ABS-KEY("neuro*") OR TITLE-ABS-KEY("cranial*) **(5,055,724 results)**
5. #2 OR #3 **(3,104,347 results)**
6. #1 AND #4 AND #5 **(4,236 results)**
7. #6 AND (EXCLUDE(DOCTYPE, "cp")) **(4,169 results)**
8. #7 AND LIMIT-TO(EXACTKEYWORD, "Incidental Finding")) **(1,966 results)**

Supplementary Figure I

Forest plots depicting the findings of each study for each category.

Aneurysm

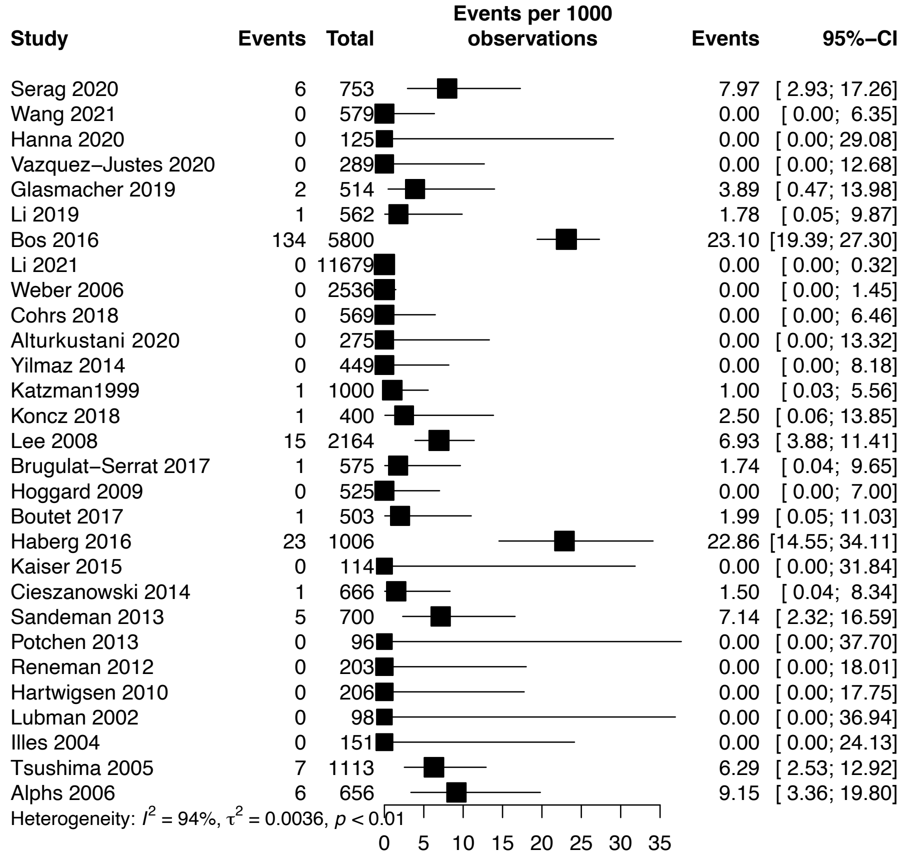

Cavernoma

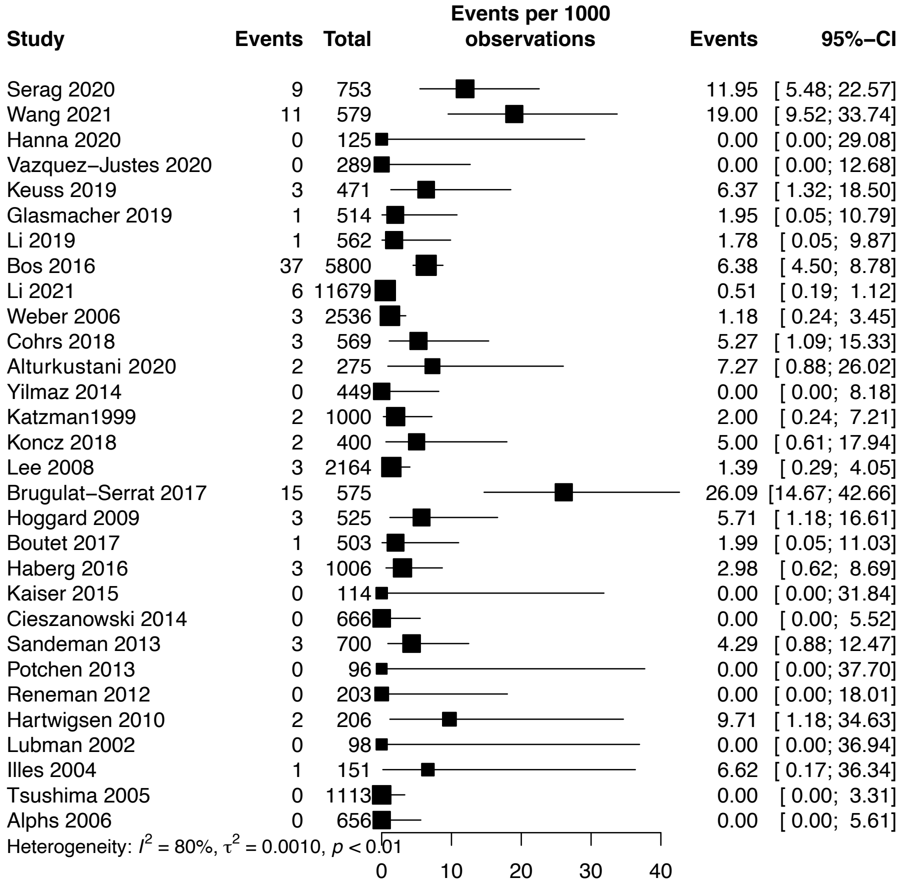

Other vascular

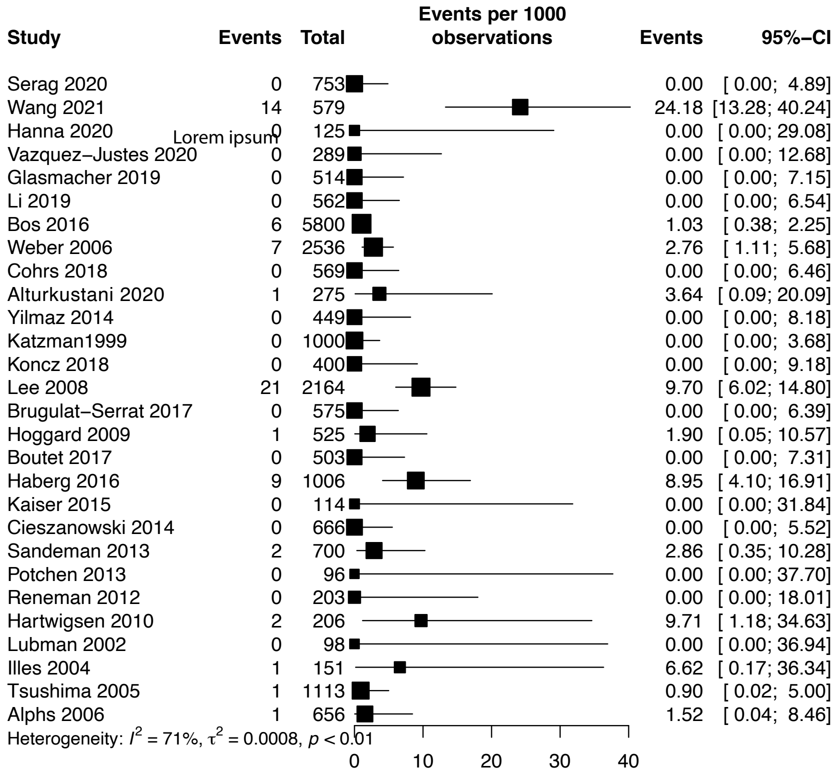

Any vascular

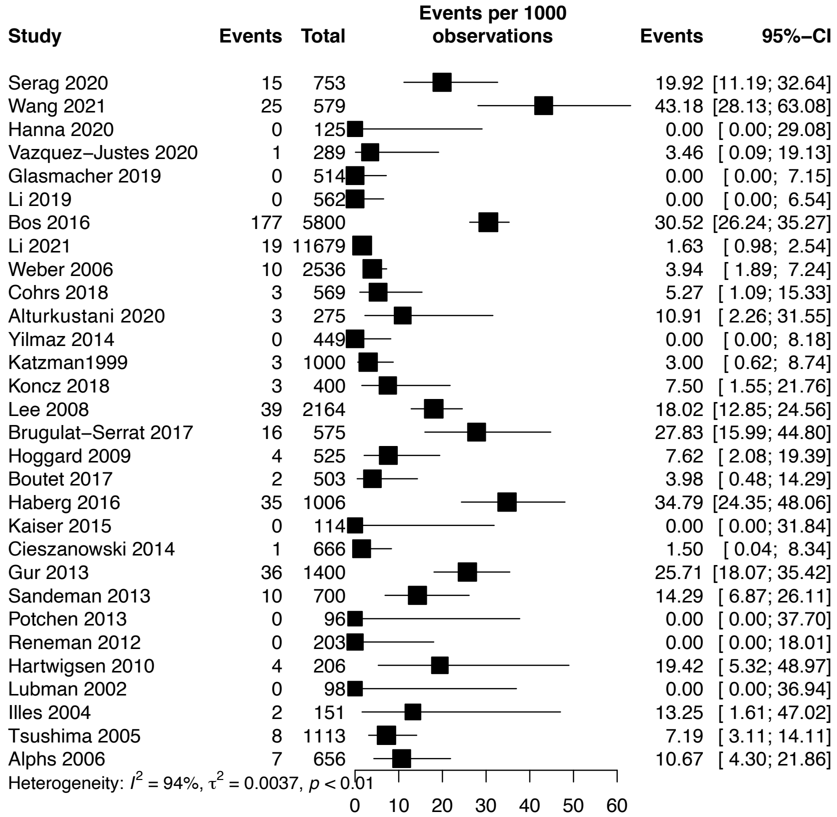

Meningioma

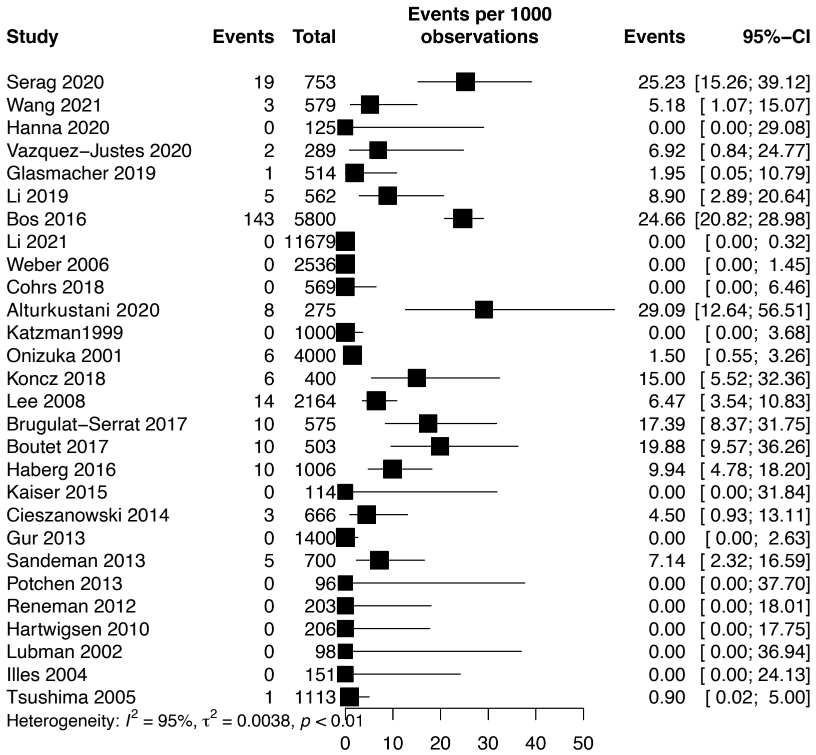

Pituitary

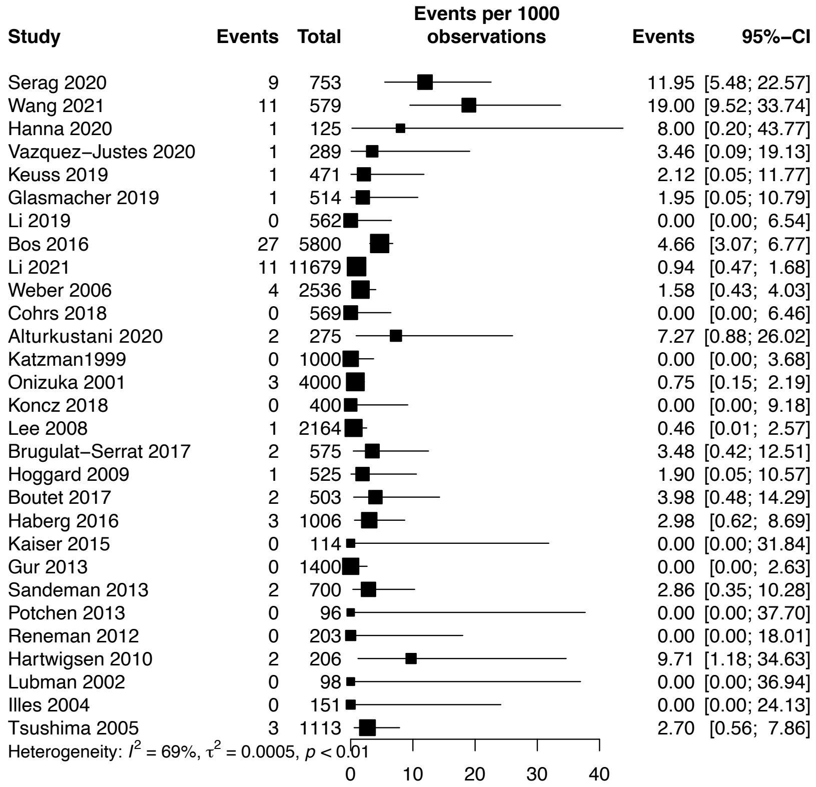

Glioma

Study	Events	Total	Events per 1000 observations	Events	95%-CI
Serag 2020	0	753		0.00	[0.00; 4.89]
Wang 2021	0	579		0.00	[0.00; 6.35]
Hanna 2020	0	125		0.00	[0.00; 29.08]
Vazquez–Justes 2020	0	289		0.00	[0.00; 12.68]
Keuss 2019	0	471		0.00	[0.00; 7.80]
Glasmacher 2019	1	514		1.95	[0.05; 10.79]
Li 2019	0	562		0.00	[0.00; 6.54]
Bos 2016	6	5800		1.03	[0.38; 2.25]
Li 2021	34	11679		2.91	[2.02; 4.07]
Weber 2006	1	2536		0.39	[0.01; 2.20]
Cohrs 2018	2	569		3.51	[0.43; 12.64]
Alturkustani 2020	1	275		3.64	[0.09; 20.09]
Katzman1999	3	1000		3.00	[0.62; 8.74]
Onizuka 2001	1	4000		0.25	[0.01; 1.39]
Koncz 2018	1	400		2.50	[0.06; 13.85]
Lee 2008	0	2164		0.00	[0.00; 1.70]
Haberg 2016	1	1006		0.99	[0.03; 5.53]
Kaiser 2015	0	114		0.00	[0.00; 31.84]
Cieszanowski 2014	1	666		1.50	[0.04; 8.34]
Gur 2013	0	1400		0.00	[0.00; 2.63]
Sandeman 2013	0	700		0.00	[0.00; 5.26]
Potchen 2013	0	96		0.00	[0.00; 37.70]
Reneman 2012	0	203		0.00	[0.00; 18.01]
Hartwigsen 2010	0	206		0.00	[0.00; 17.75]
Lubman 2002	0	98		0.00	[0.00; 36.94]
Illes 2004	0	151		0.00	[0.00; 24.13]
Tsushima 2005	0	1113		0.00	[0.00; 3.31]

Heterogeneity: $I^2 = 40\%$, $\tau^2 = 0.0001$, $p = 0.02$

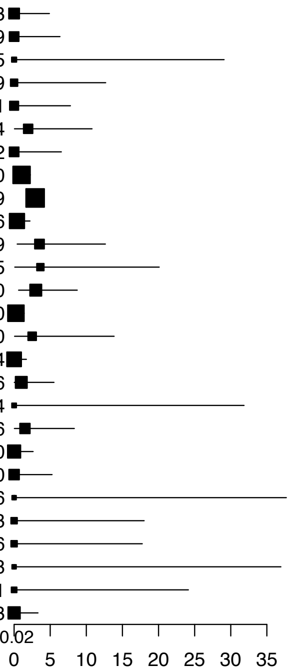

Other neoplasm

Study	Events	Total	Events per 1000 observations	Events	95%-CI
Serag 2020	11	753		14.61	[7.31; 25.99]
Wang 2021	4	579		6.91	[1.89; 17.59]
Hanna 2020	0	125		0.00	[0.00; 29.08]
Vazquez–Justes 2020	2	289		6.92	[0.84; 24.77]
Glasmacher 2019	1	514		1.95	[0.05; 10.79]
Li 2019	0	562		0.00	[0.00; 6.54]
Bos 2016	11	5800		1.90	[0.95; 3.39]
Li 2021	0	11679		0.00	[0.00; 0.32]
Weber 2006	4	2536		1.58	[0.43; 4.03]
Cohrs 2018	0	569		0.00	[0.00; 6.46]
Alturkustani 2020	1	275		3.64	[0.09; 20.09]
Yilmaz 2014	1	449		2.23	[0.06; 12.35]
Katzman1999	1	1000		1.00	[0.03; 5.56]
Onizuka 2001	0	4000		0.00	[0.00; 0.92]
Koncz 2018	7	400		17.50	[7.06; 35.72]
Lee 2008	0	2164		0.00	[0.00; 1.70]
Brugulat–Serrat 2017	2	575		3.48	[0.42; 12.51]
Hoggard 2009	4	525		7.62	[2.08; 19.39]
Boutet 2017	2	503		3.98	[0.48; 14.29]
Haberg 2016	0	1006		0.00	[0.00; 3.66]
Kaiser 2015	2	114		17.54	[2.13; 61.94]
Gur 2013	0	1400		0.00	[0.00; 2.63]
Sandeman 2013	3	700		4.29	[0.88; 12.47]
Potchen 2013	0	96		0.00	[0.00; 37.70]
Reneman 2012	0	203		0.00	[0.00; 18.01]
Hartwigsen 2010	0	206		0.00	[0.00; 17.75]
Lubman 2002	1	98		10.20	[0.26; 55.54]
Illes 2004	0	151		0.00	[0.00; 24.13]
Tsushima 2005	1	1113		0.90	[0.02; 5.00]
Alphs 2006	6	656		9.15	[3.36; 19.80]

Heterogeneity: $I^2 = 81\%$, $\tau^2 = 0.0009$, $p < 0.01$

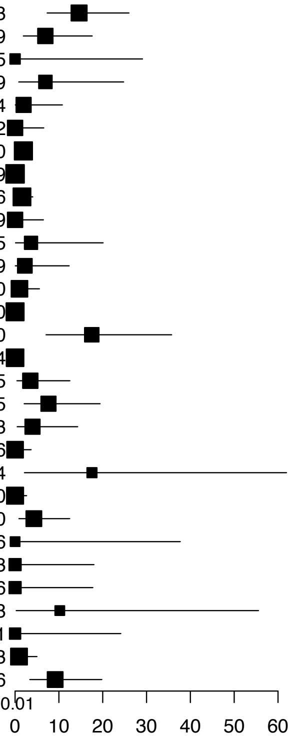

Any neoplasm

Study	Events	Total	Events per 1000 observations	Events	95%-CI
Serag 2020	39	753		51.79	[37.09; 70.13]
Wang 2021	18	579		31.09	[18.53; 48.69]
Hanna 2020	1	125		8.00	[0.20; 43.77]
Vazquez–Justes 2020	5	289		17.30	[5.64; 39.91]
Glasmacher 2019	6	514		11.67	[4.30; 25.23]
Li 2019	5	562		8.90	[2.89; 20.64]
Bos 2016	195	5800		33.62	[29.13; 38.59]
Li 2021	45	11679		3.85	[2.81; 5.15]
Weber 2006	9	2536		3.55	[1.62; 6.73]
Cohrs 2018	2	569		3.51	[0.43; 12.64]
Alturkustani 2020	12	275		43.64	[22.75; 74.98]
Yilmaz 2014	1	449		2.23	[0.06; 12.35]
Katzman1999	3	1000		3.00	[0.62; 8.74]
Onizuka 2001	10	4000		2.50	[1.20; 4.59]
Koncz 2018	14	400		35.00	[19.26; 58.03]
Rocque 2008	16	2164		7.39	[4.23; 11.98]
Brugulat–Serrat 2017	14	575		24.35	[13.37; 40.51]
Hoggard 2009	5	525		9.52	[3.10; 22.08]
Boutet 2017	15	503		29.82	[16.78; 48.71]
Haberg 2016	15	1006		14.91	[8.37; 24.47]
Kaiser 2015	2	114		17.54	[2.13; 61.94]
Gur 2013	0	1400		0.00	[0.00; 2.63]
Sandeman 2013	10	700		14.29	[6.87; 26.11]
Potchen 2013	0	96		0.00	[0.00; 37.70]
Reneman 2012	0	203		0.00	[0.00; 18.01]
Hartwigsen 2010	2	206		9.71	[1.18; 34.63]
Lubman 2002	1	98		10.20	[0.26; 55.54]
Illes 2004	0	151		0.00	[0.00; 24.13]
Tsushima 2005	5	1113		4.49	[1.46; 10.45]
Alphs 2006	6	656		9.15	[3.36; 19.80]

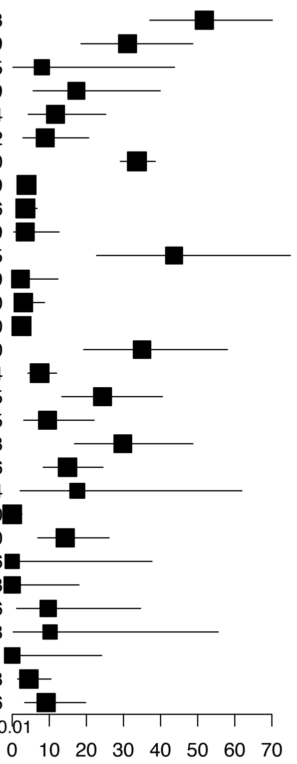

Heterogeneity: $I^2 = 94\%$, $\tau^2 = 0.0033$, $p < 0.01$

Chiari malformation

Study	Events	Total	Events per 1000 observations	Events	95%-CI
Serag 2020	6	753		7.97	[2.93; 17.26]
Wang 2021	0	579		0.00	[0.00; 6.35]
Hanna 2020	1	125		8.00	[0.20; 43.77]
Vazquez–Justes 2020	1	289		3.46	[0.09; 19.13]
Glasmacher 2019	0	514		0.00	[0.00; 7.15]
Li 2019	0	562		0.00	[0.00; 6.54]
Li 2021	23	11679		1.97	[1.25; 2.95]
Weber 2006	43	2536		16.96	[12.30; 22.77]
Cohrs 2018	0	569		0.00	[0.00; 6.46]
Alturkustani 2020	1	275		3.64	[0.09; 20.09]
Yilmaz 2014	3	449		6.68	[1.38; 19.40]
Katzman1999	0	1000		0.00	[0.00; 3.68]
Koncz 2018	0	400		0.00	[0.00; 9.18]
Lee 2008	0	2164		0.00	[0.00; 1.70]
Brugulat–Serrat 2017	6	575		10.43	[3.84; 22.57]
Hoggard 2009	0	525		0.00	[0.00; 7.00]
Boutet 2017	0	503		0.00	[0.00; 7.31]
Haberg 2016	2	1006		1.99	[0.24; 7.16]
Kaiser 2015	2	114		17.54	[2.13; 61.94]
SoeMar 2013	16	375		42.67	[24.58; 68.36]
Sandeman 2013	1	700		1.43	[0.04; 7.93]
Potchen 2013	0	96		0.00	[0.00; 37.70]
Reneman 2012	2	203		9.85	[1.20; 35.13]
Hartwigsen 2010	2	206		9.71	[1.18; 34.63]
Lubman 2002	0	98		0.00	[0.00; 36.94]
Tsushima 2005	0	1113		0.00	[0.00; 3.31]

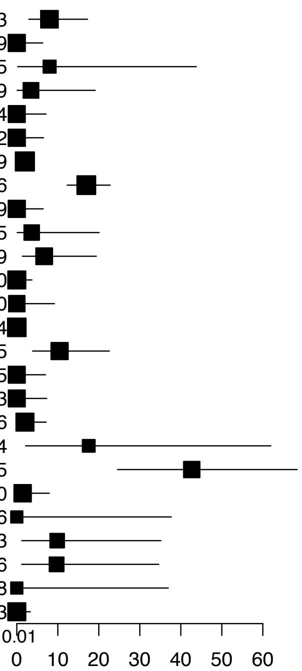

Heterogeneity: $I^2 = 85\%$, $\tau^2 = 0.0017$, $p < 0.01$

Pineal cyst

Study	Events	Total	Events per 1000 observations	Events	95%-CI
Serag 2020	0	753		0.00	[0.00; 4.89]
Wang 2021	4	579		6.91	[1.89; 17.59]
Hanna 2020	0	125		0.00	[0.00; 29.08]
Vazquez−Justes 2020	0	289		0.00	[0.00; 12.68]
Keuss 2019	0	471		0.00	[0.00; 7.80]
Glasmacher 2019	0	514		0.00	[0.00; 7.15]
Li 2019	0	562		0.00	[0.00; 6.54]
Vernooij 2007	0	2000		0.00	[0.00; 1.84]
Li 2021	910	11679		77.92	[73.12; 82.93]
Weber 2006	0	2536		0.00	[0.00; 1.45]
Cohrs 2018	4	569		7.03	[1.92; 17.90]
Alturkustani 2020	0	275		0.00	[0.00; 13.32]
Yilmaz 2014	1	449		2.23	[0.06; 12.35]
Kim 2002	1	225		4.44	[0.11; 24.51]
Katzman1999	2	1000		2.00	[0.24; 7.21]
Koncz 2018	0	400		0.00	[0.00; 9.18]
Lee 2008	0	2164		0.00	[0.00; 1.70]
Brugulat−Serrat 2017	2	575		3.48	[0.42; 12.51]
Hoggard 2009	0	525		0.00	[0.00; 7.00]
Boutet 2017	168	503		334.00	[292.87; 377.09]
Haberg 2016	1	1006		0.99	[0.03; 5.53]
Kaiser 2015	2	114		17.54	[2.13; 61.94]
Cieszanowski 2014	12	666		18.02	[9.34; 31.26]
Gur 2013	34	1400		24.29	[16.88; 33.77]
SoeMar 2013	10	375		26.67	[12.86; 48.49]
Sandeman 2013	0	700		0.00	[0.00; 5.26]
Potchen 2013	0	96		0.00	[0.00; 37.70]
Reneman 2012	12	203		59.11	[30.91; 100.98]
Hartwigsen 2010	12	206		58.25	[30.46; 99.54]
Lubman 2002	1	98		10.20	[0.26; 55.54]
Tsushima 2005	0	1113		0.00	[0.00; 3.31]

Heterogeneity: $I^2 = 99\%$, $\tau^2 = 0.0211$, $p = 0$

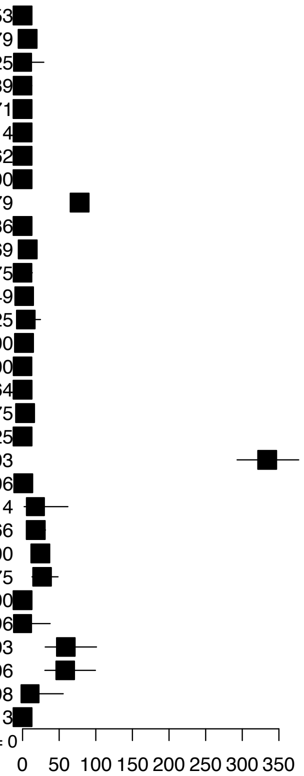

Arachnoid cyst

Study	Events	Total	Events per 1000 observations	Events	95%-CI
Serag 2020	11	753		14.61	[7.31; 25.99]
Wang 2021	8	579		13.82	[5.98; 27.04]
Hanna 2020	0	125		0.00	[0.00; 29.08]
Vazquez−Justes 2020	1	289		3.46	[0.09; 19.13]
Keuss 2019	0	471		0.00	[0.00; 7.80]
Glasmacher 2019	0	514		0.00	[0.00; 7.15]
Li 2019	1	562		1.78	[0.05; 9.87]
Bos 2016	92	5800		15.86	[12.81; 19.42]
Vernooij 2007	0	2000		0.00	[0.00; 1.84]
Li 2021	128	11679		10.96	[9.15; 13.02]
Weber 2006	43	2536		16.96	[12.30; 22.77]
Cohrs 2018	8	569		14.06	[6.09; 27.51]
Alturkustani 2020	0	275		0.00	[0.00; 13.32]
Yilmaz 2014	2	449		4.45	[0.54; 16.00]
Kim 2002	2	225		8.89	[1.08; 31.74]
Katzman1999	3	1000		3.00	[0.62; 8.74]
Koncz 2018	3	400		7.50	[1.55; 21.76]
Lee 2008	15	2164		6.93	[3.88; 11.41]
Brugulat−Serrat 2017	3	575		5.22	[1.08; 15.17]
Hoggard 2009	1	525		1.90	[0.05; 10.57]
Boutet 2017	16	503		31.81	[18.29; 51.14]
Haberg 2016	36	1006		35.79	[25.19; 49.20]
Kaiser 2015	0	114		0.00	[0.00; 31.84]
Cieszanowski 2014	10	666		15.02	[7.22; 27.44]
SoeMar 2013	14	375		37.33	[20.56; 61.85]
Sandeman 2013	10	700		14.29	[6.87; 26.11]
Potchen 2013	2	96		20.83	[2.53; 73.24]
Hartwigsen 2010	0	206		0.00	[0.00; 17.75]
Lubman 2002	0	98		0.00	[0.00; 36.94]
Tsushima 2005	5	1113		4.49	[1.46; 10.45]

Heterogeneity: $I^2 = 87\%$, $\tau^2 = 0.0016$, $p < 0.01$

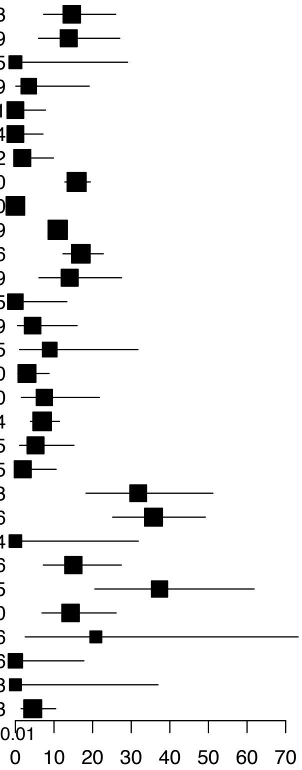

Supplementary Table I
Results of univariable meta-regressions for each analysis.

Var	Analysis	Studies	N	β	95%CI	R^2	p-value
Age	Any neoplastic	30	39,040	-0.000316	(0.000701 - 0.00234)	36.72	0.079
Male	Any neoplastic	30	39,040	-0.018000	(-0.221 - 0.0529)	2.16	0.53
Age	Any vascular	30	35,706	0.001680	(8.02e-05 - 0.00189)	12.84	<0.001
Male	Any vascular	30	35,706	-0.140000	(-0.17 - 0.0935)	0.00	0.059
Age	Aneurysm	29	34,306	0.000499	(0.000531 - 0.00184)	38.93	0.12
Male	Aneurysm	29	34,306	0.020900	(-0.114 - 0.0924)	0.00	0.65
Age	Arachnoid cyst	30	36,367	0.000232	(-0.000995 - 0.000706)	0.00	0.53
Male	Arachnoid cyst	30	36,367	-0.010100	(-0.135 - 0.157)	0.00	0.82
Age	Cavernoma	30	34,777	-0.000610	(-0.000122 - 0.00118)	10.20	0.55
Male	Cavernoma	30	34,777	-0.262000	(-0.174 - -0.0102)	19.03	0.14
Age	Chiari malformation	26	27,408	-0.000561	(-0.00184 - -0.00019)	18.30	0.032
Male	Chiari malformation	26	27,408	-0.050300	(-0.152 - 0.145)	0.00	0.28
Age	Glioma	27	37,469	-0.000316	(-0.000669 - 3.7e-05)	25.62	0.079
Male	Glioma	27	37,469	-0.018000	(-0.0746 - 0.0386)	0.00	0.53
Age	Meningioma	28	38,076	0.001680	(0.000961 - 0.0024)	51.64	<0.001
Male	Meningioma	28	38,076	-0.140000	(-0.286 - 0.00513)	11.77	0.059
Age	Other neoplastic	30	39,040	0.000499	(-0.000136 - 0.00113)	10.39	0.12
Male	Other neoplastic	30	39,040	0.020900	(-0.0705 - 0.112)	0.00	0.65
Age	Other vascular	28	22,627	0.000232	(-0.00049 - 0.000954)	0.00	0.53
Male	Other vascular	28	22,627	-0.010100	(-0.0963 - 0.0761)	0.00	0.82
Age	Pineal cyst	31	32,170	-0.000610	(-0.0026 - 0.00138)	0.00	0.55
Male	Pineal cyst	31	32,170	-0.262000	(-0.612 - 0.0879)	3.91	0.14
Age	Pituitary	29	38,406	0.000561	(4.95e-05 - 0.00107)	26.38	0.032
Male	Pituitary	29	38,406	-0.050300	(-0.141 - 0.0407)	0.00	0.28

Supplementary Table II

Results of multivariable meta-regressions for each analysis.

Var	Analysis	Studies	N	β	95%CI	R^2	p-value
Age	Any neoplastic	30	39,040	0.001500	(0.000692 - 0.00231)	38.22	<0.001
Male	Any neoplastic	30	39,040	-0.078400	(-0.191 - 0.0338)		0.17
Age	Any vascular	30	35,706	0.000983	(7.03e-05 - 0.0019)	11.03	0.035
Male	Any vascular	30	35,706	-0.039100	(-0.163 - 0.0853)		0.54
Age	Aneurysm	29	34,306	0.001180	(0.000509 - 0.00185)	35.96	<0.001
Male	Aneurysm	29	34,306	-0.004390	(-0.0898 - 0.0811)		0.92
Age	Arachnoid cyst	30	36,367	-0.000140	(-0.00101 - 0.000731)	0.00	0.75
Male	Arachnoid cyst	30	36,367	0.008380	(-0.141 - 0.157)		0.91
Age	Cavernoma	30	34,777	0.000470	(-0.000142 - 0.00108)	25.42	0.13
Male	Cavernoma	30	34,777	-0.085900	(-0.166 - -0.00623)		0.035
Age	Chiari malformation	26	27,408	-0.001030	(-0.00188 - -0.000179)	12.87	0.018
Male	Chiari malformation	26	27,408	-0.017000	(-0.154 - 0.12)		0.81
Age	Glioma	27	37,469	-0.000378	(-0.000715 - -3.99e-05)	45.24	0.028
Male	Glioma	27	37,469	-0.033000	(-0.083 - 0.0169)		0.19
Age	Meningioma	28	38,076	0.001560	(0.000838 - 0.00228)	54.23	<0.001
Male	Meningioma	28	38,076	-0.091600	(-0.204 - 0.021)		0.11
Age	Other neoplastic	30	39,040	0.000509	(-0.000132 - 0.00115)	8.49	0.12
Male	Other neoplastic	30	39,040	0.025800	(-0.0615 - 0.113)		0.56
Age	Other vascular	28	22,627	0.000222	(-0.000521 - 0.000966)	0.00	0.56
Male	Other vascular	28	22,627	-0.006650	(-0.0945 - 0.0812)		0.88
Age	Pineal cyst	31	32,170	-0.000603	(-0.00255 - 0.00135)	1.82	0.54
Male	Pineal cyst	31	32,170	-0.261000	(-0.615 - 0.0922)		0.15
Age	Pituitary	29	38,406	0.000511	(-3.79e-05 - 0.00106)	17.99	0.068
Male	Pituitary	29	38,406	-0.027100	(-0.114 - 0.0597)		0.54

Supplementary Figure II

Relationship between proportions for each finding and age in each analysis, containing studies in healthy volunteers only. Points represent the findings of the individual studies, with size of the point proportional to the sample size of the study. The black line represents the fitted restricted cubic spline model, and the shaded area its 95% confidence interval.

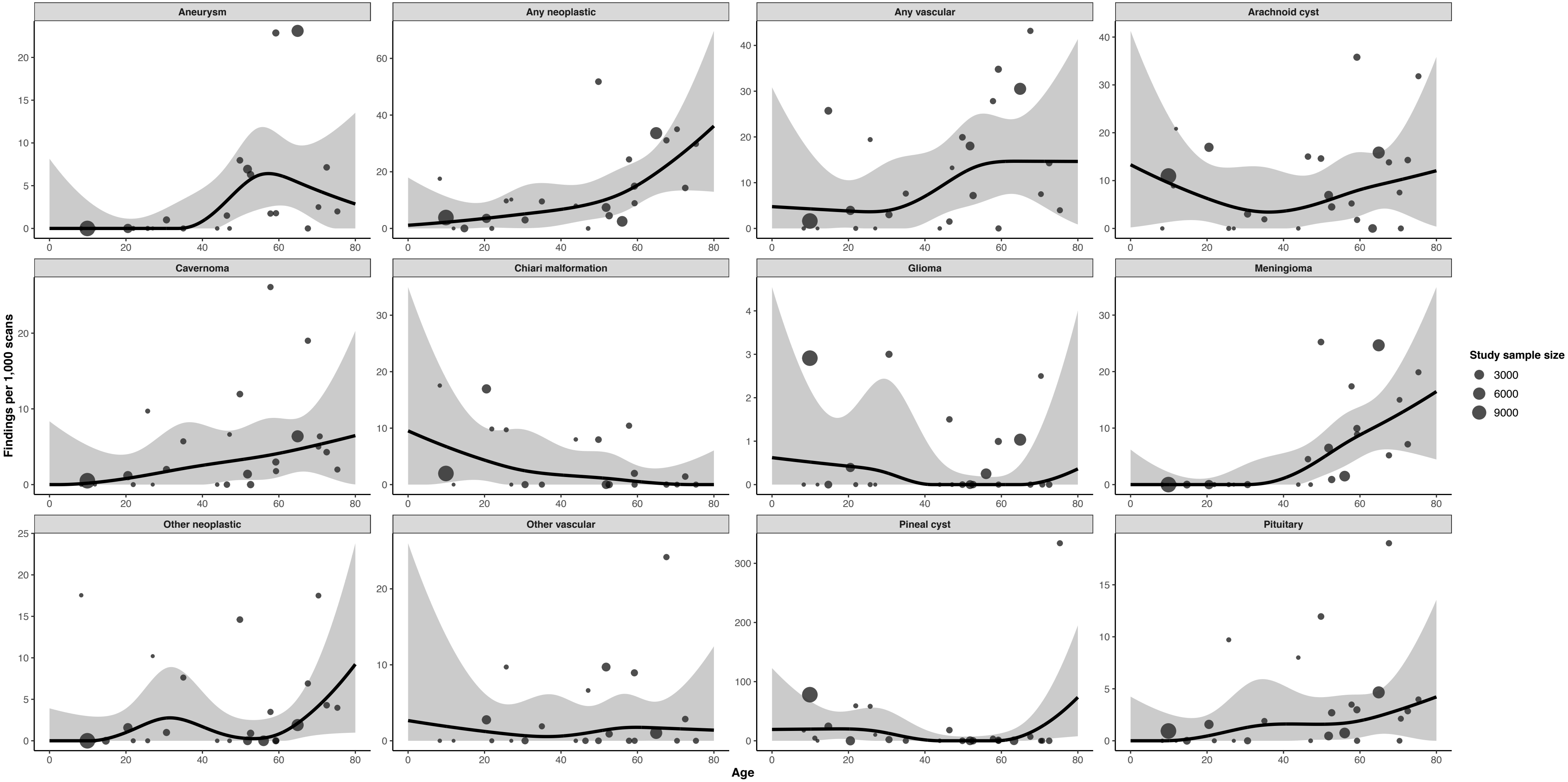

Supplementary Table III

Age-stratified estimates of proportions for each finding from restricted cubic spline models, limited to studies in healthy volunteers only.

	**Age - Findings /1,000 scans (95%CI)**									
**Finding**	**1**	**5**	**10**	**20**	**30**	**40**	**50**	**60**	**70**	**80**
Vascular										
Aneurysm	0 (0 - 8)	0 (0 - 5)	0 (0 - 3)	0 (0 - 1)	0 (0 - 2)	0.8 (0 - 5)	5 (1 - 10)	6 (3 - 11)	4 (0.8 - 10)	3 (0 - 14)
Cavernoma	0 (0 - 8)	0.03 (0 - 7)	0.2 (0 - 5)	0.8 (0 - 4)	2 (0 - 7)	3 (0.001 - 8)	3 (0.6 - 7)	4 (1 - 9)	5 (1 - 11)	6 (0.02 - 20)
Other vascular	3 (0 - 25)	2 (0 - 19)	2 (0 - 14)	1 (0 - 6)	0.7 (0 - 5)	0.6 (0 - 6)	1 (0 - 5)	2 (0 - 6)	2 (0 - 6)	1 (0 - 12)
Any vascular	5 (0 - 29)	5 (0 - 23)	4 (0 - 17)	4 (0.2 - 11)	4 (0 - 14)	7 (1 - 17)	12 (5 - 23)	15 (7 - 24)	15 (5 - 28)	15 (0.9 - 41)
Neoplastic										
Meningioma	0 (0 - 6)	0 (0 - 4)	0 (0 - 2)	0 (0 - 1)	0.0009 (0 - 4)	1 (0 - 6)	5 (1 - 9)	9 (5 - 14)	12 (6 - 21)	16 (4 - 35)
Pituitary	0 (0 - 4)	0 (0 - 3)	0.007 (0 - 3)	0.5 (0 - 2)	1 (0 - 5)	2 (0 - 5)	2 (0.1 - 4)	2 (0.3 - 4)	3 (0.5 - 7)	4 (0 - 14)
Glioma	0.6 (0 - 4)	0.6 (0 - 3)	0.5 (0 - 2)	0.4 (0 - 2)	0.3 (0 - 2)	0.01 (0 - 1)	0 (0 - 0.2)	0 (0 - 0.2)	0.05 (0 - 1)	0.4 (0 - 4)
Other neoplastic	0 (0 - 4)	0 (0 - 3)	0 (0 - 3)	1 (0 - 4)	3 (0 - 9)	2 (0 - 6)	0.4 (0 - 3)	0.8 (0 - 3)	4 (0.7 - 9)	9 (1 - 24)
Any neoplastic	1 (0 - 17)	2 (0 - 14)	2 (0 - 11)	4 (0.3 - 9)	5 (0.09 - 15)	7 (1 - 16)	9 (4 - 18)	15 (8 - 24)	25 (13 - 39)	36 (13 - 70)
Chiari malformation	9 (0 - 33)	8 (0.003 - 26)	7 (0.3 - 19)	4 (0.7 - 10)	3 (0 - 10)	2 (0 - 7)	1 (0 - 6)	0.6 (0 - 3)	0.07 (0 - 3)	0 (0 - 6)
Pineal cyst	19 (0 - 116)	19 (0 - 92)	20 (0.0007 - 67)	20 (3 - 50)	13 (0 - 52)	2 (0 - 19)	0 (0 - 7)	0.4 (0 - 13)	22 (2 - 60)	74 (8 - 195)
Arachnoid cyst	13 (0.3 - 39)	11 (0.7 - 31)	10 (1 - 23)	6 (1 - 14)	4 (0 - 13)	4 (0 - 12)	5 (1 - 12)	8 (3 - 16)	10 (4 - 19)	12 (0.4 - 36)

Supplementary Figure III

Relationship between age and proportion of findings derived from conventional linear regression models.

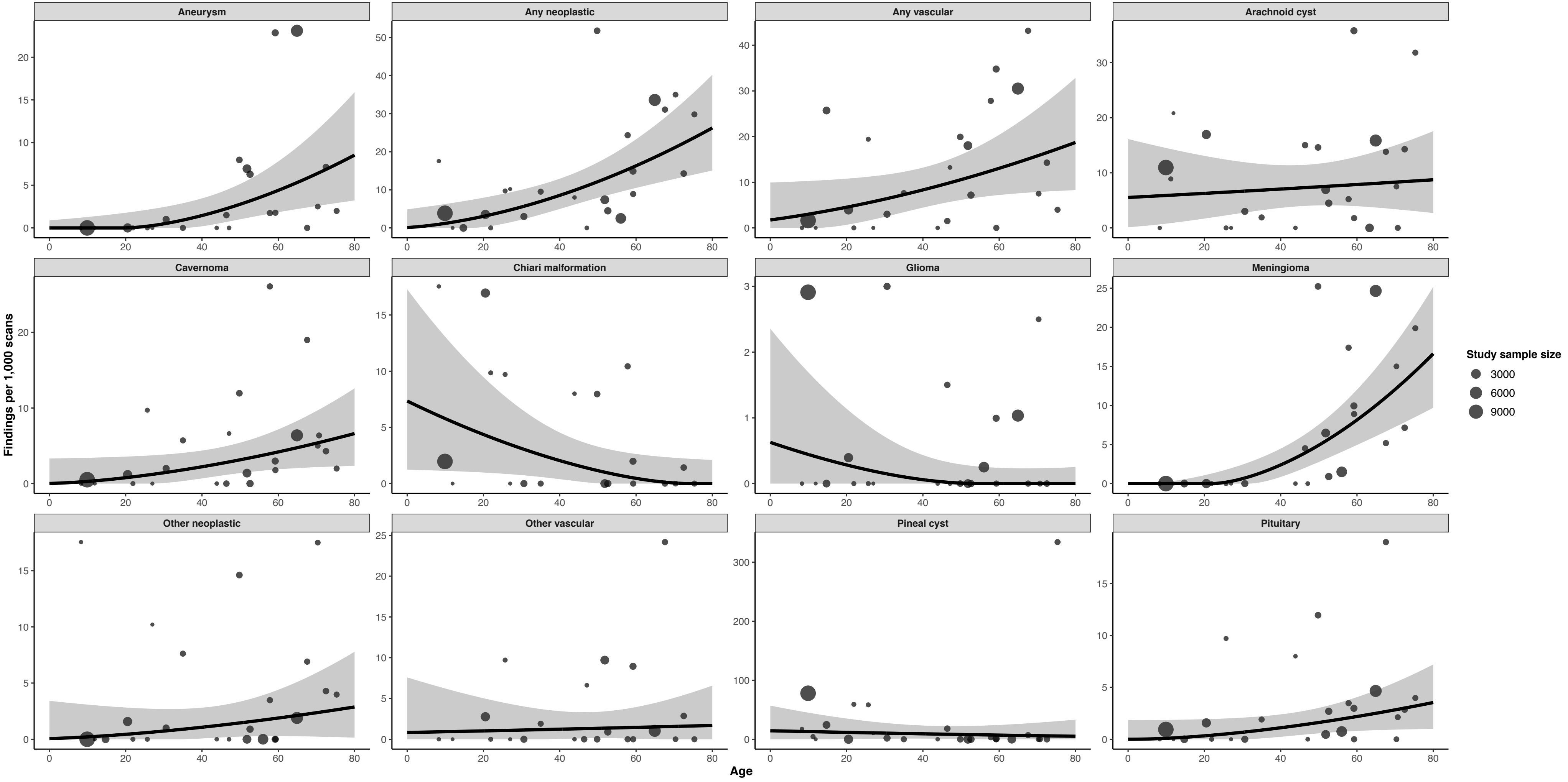

Supplementary Table IV

Age-stratified estimates of proportions, derived from univariable linear regression models.

	**Age - Findings /1,000 scans (95%CI)**									
**Finding**	**1**	**5**	**10**	**20**	**30**	**40**	**50**	**60**	**70**	**80**
Vascular										
Aneurysm	0 (0 - 0.9)	0 (0 - 1)	0 (0 - 1)	0.005 (0 - 2)	0.5 (0 - 2)	1 (0.2 - 3)	3 (1 - 5)	4 (2 - 8)	6 (3 - 11)	9 (3 - 16)
Cavernoma	0.03 (0 - 3)	0.1 (0 - 3)	0.3 (0 - 3)	0.8 (0 - 4)	1 (0.07 - 4)	2 (0.7 - 4)	3 (1 - 5)	4 (2 - 7)	5 (2 - 10)	7 (2 - 13)
Other vascular	0.8 (0 - 7)	0.9 (0 - 7)	0.9 (0 - 6)	1 (0 - 5)	1 (0 - 4)	1 (0.04 - 3)	1 (0.1 - 3)	1 (0.07 - 4)	2 (0 - 5)	2 (0 - 7)
Any vascular	2 (0 - 10)	2 (0 - 10)	3 (0 - 10)	5 (0.7 - 11)	6 (2 - 12)	8 (4 - 13)	11 (6 - 16)	13 (7 - 20)	16 (8 - 26)	19 (8 - 33)
Neoplastic										
Meningioma	0 (0 - 0)	0 (0 - 0.02)	0 (0 - 0.2)	0 (0 - 1)	0.6 (0 - 2)	2 (0.8 - 4)	5 (3 - 8)	8 (5 - 12)	12 (7 - 18)	17 (10 - 25)
Pituitary	0.0004 (0 - 2)	0.02 (0 - 2)	0.09 (0 - 2)	0.3 (0 - 2)	0.7 (0.002 - 2)	1 (0.2 - 2)	2 (0.6 - 3)	2 (0.8 - 4)	3 (0.9 - 5)	4 (1 - 7)
Glioma	0.6 (0 - 2)	0.5 (0 - 2)	0.4 (0 - 2)	0.3 (0 - 1)	0.2 (0 - 0.7)	0.06 (0 - 0.4)	0.007 (0 - 0.3)	0 (0 - 0.2)	0 (0 - 0.2)	0 (0 - 0.3)
Other neoplastic	0.07 (0 - 3)	0.1 (0 - 3)	0.2 (0 - 3)	0.4 (0 - 3)	0.7 (0 - 3)	1 (0.07 - 3)	1 (0.2 - 3)	2 (0.3 - 4)	2 (0.2 - 6)	3 (0.1 - 8)
Any neoplastic	0.2 (0 - 5)	0.5 (0 - 6)	1 (0 - 6)	3 (0.2 - 8)	6 (2 - 10)	9 (5 - 13)	12 (8 - 17)	16 (11 - 23)	21 (13 - 31)	26 (15 - 40)
Chiari malformation	7 (1 - 17)	7 (1 - 15)	6 (1 - 13)	4 (1 - 9)	3 (0.8 - 7)	2 (0.4 - 5)	1 (0.03 - 3)	0.5 (0 - 3)	0.05 (0 - 2)	0 (0 - 2)
Pineal cyst	14 (0 - 56)	14 (0 - 51)	13 (0 - 45)	12 (0.3 - 35)	10 (0.9 - 28)	9 (1 - 23)	8 (0.5 - 22)	7 (0 - 24)	6 (0 - 28)	5 (0 - 33)
Arachnoid cyst	6 (0.2 - 16)	6 (0.4 - 15)	6 (0.8 - 14)	6 (2 - 13)	7 (3 - 12)	7 (4 - 11)	7 (4 - 12)	8 (4 - 13)	8 (3 - 15)	9 (3 - 18)

Supplementary Figure IV

Relationship between age and proportions for each finding, derived from linear regression models adjusted for both age and gender. Models are fitted with gender proportion held at an equal number of males and females.

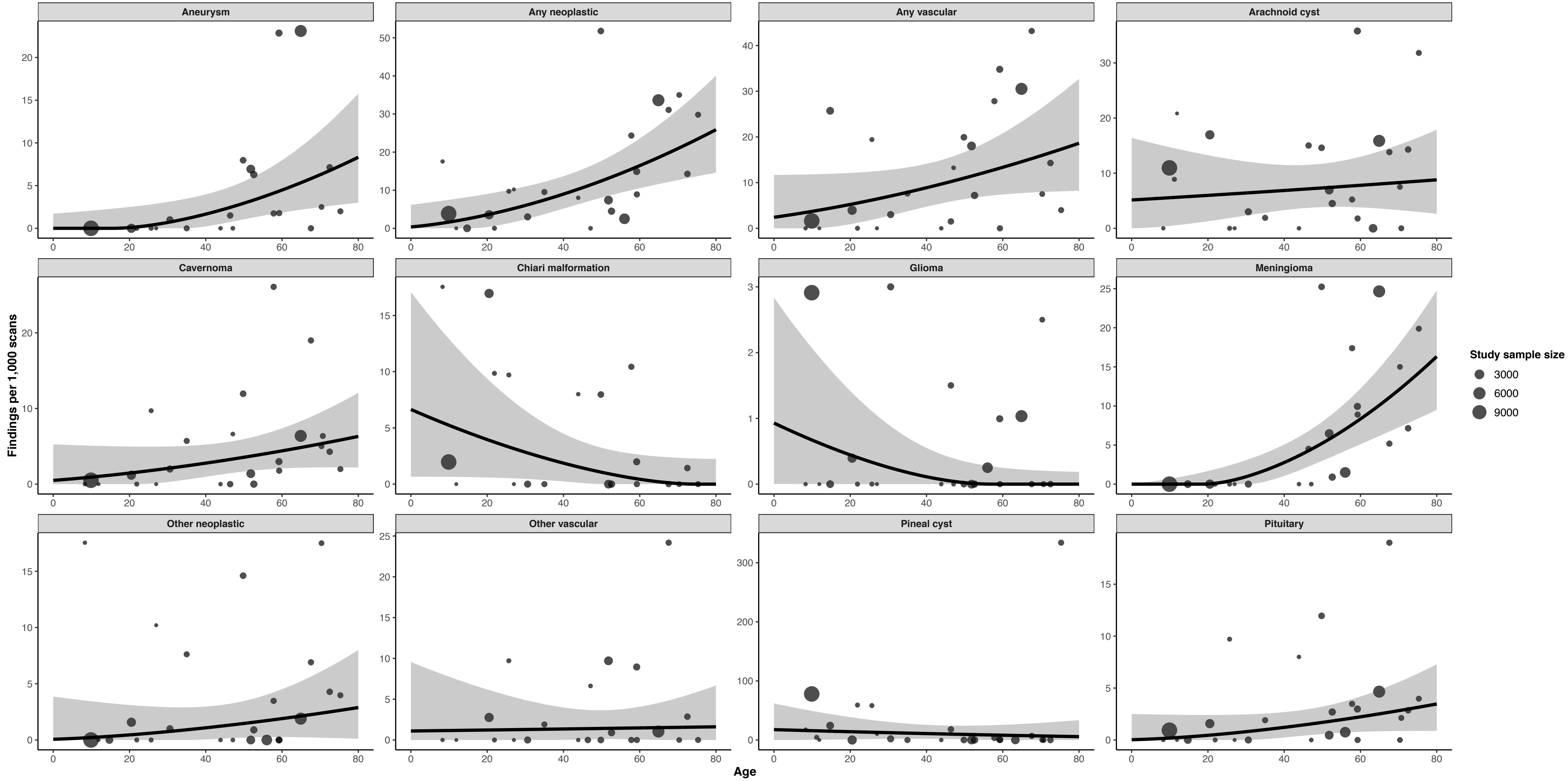

Supplementary Table V

Age-stratified estimates of proportions for each finding, derived from multivariable linear regression models adjusted for age and gender proportion. Estimates are derived from predictions assuming each and an equal distribution of males and females.

	**Age - Findings /1,000 scans (95%CI)**									
**Finding**	**1**	**5**	**10**	**20**	**30**	**40**	**50**	**60**	**70**	**80**
Vascular										
Aneurysm	0 (0 - 2)	0 (0 - 2)	0 (0 - 2)	0.08 (0 - 3)	0.7 (0 - 3)	2 (0.2 - 4)	3 (1 - 5)	4 (2 - 8)	6 (2 - 11)	8 (3 - 16)
Cavernoma	0.5 (0 - 5)	0.7 (0 - 5)	0.9 (0 - 5)	1 (0 - 5)	2 (0.3 - 5)	3 (1 - 5)	4 (2 - 6)	4 (2 - 7)	5 (2 - 9)	6 (2 - 12)
Other vascular	1 (0 - 9)	1 (0 - 9)	1 (0 - 8)	1 (0 - 6)	1 (0 - 5)	1 (0.01 - 4)	1 (0.1 - 4)	1 (0.06 - 4)	2 (0 - 5)	2 (0 - 7)
Any vascular	3 (0 - 12)	3 (0 - 12)	4 (0 - 12)	5 (0.9 - 12)	7 (3 - 13)	9 (5 - 14)	11 (6 - 17)	13 (7 - 21)	16 (8 - 26)	19 (8 - 33)
						2 (0.2 - 4)	3 (1 - 5)	4 (2 - 8)	6 (2 - 11)	8 (3 - 16)
Neoplastic										
Meningioma	0 (0 - 0.1)	0 (0 - 0.3)	0 (0 - 0.7)	0.02 (0 - 2)	1 (0 - 3)	3 (1 - 5)	5 (3 - 8)	8 (5 - 12)	12 (7 - 18)	16 (9 - 25)
Pituitary	0.04 (0 - 2)	0.09 (0 - 2)	0.2 (0 - 2)	0.5 (0 - 2)	0.8 (0.002 - 2)	1 (0.2 - 3)	2 (0.6 - 3)	2 (0.8 - 4)	3 (0.9 - 6)	3 (0.9 - 7)
Glioma	0.9 (0.003 - 3)	0.8 (0.003 - 2)	0.7 (0.002 - 2)	0.4 (0.0004 - 1)	0.3 (0 - 0.9)	0.1 (0 - 0.6)	0.02 (0 - 0.3)	0 (0 - 0.2)	0 (0 - 0.2)	0 (0 - 0.2)
Other neoplastic	0.07 (0 - 4)	0.1 (0 - 4)	0.2 (0 - 3)	0.5 (0 - 3)	0.7 (0 - 3)	1 (0.04 - 3)	1 (0.2 - 3)	2 (0.3 - 5)	2 (0.2 - 6)	3 (0.1 - 8)
Any neoplastic	0.4 (0 - 6)	0.9 (0 - 7)	2 (0 - 7)	4 (0.3 - 9)	6 (2 - 11)	9 (5 - 14)	12 (8 - 18)	16 (11 - 24)	21 (13 - 31)	26 (15 - 40)
Chiari malformation	6 (0.7 - 17)	6 (0.7 - 15)	5 (0.6 - 13)	4 (0.6 - 9)	3 (0.5 - 7)	2 (0.2 - 4)	1 (0.003 - 3)	0.4 (0 - 3)	0.06 (0 - 2)	0 (0 - 2)
Pineal cyst	17 (0 - 61)	17 (0.01 - 55)	16 (0.2 - 49)	14 (1 - 39)	12 (2 - 31)	11 (2 - 26)	9 (1 - 24)	8 (0.03 - 26)	7 (0 - 29)	6 (0 - 34)
Arachnoid cyst	5 (0.01 - 16)	5 (0.1 - 16)	6 (0.4 - 15)	6 (1 - 13)	6 (2 - 12)	7 (3 - 11)	7 (4 - 12)	8 (4 - 13)	8 (3 - 15)	9 (3 - 18)

Supplementary Figure V
Funnel plots for each analysis. Funnel plots are plots of sample size versus log odds, as conventional funnel plots may be inaccurate in estimates of rare proportions.

Vascular findings

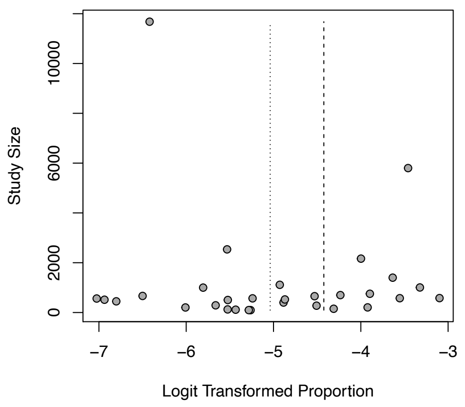

Neoplastic findings

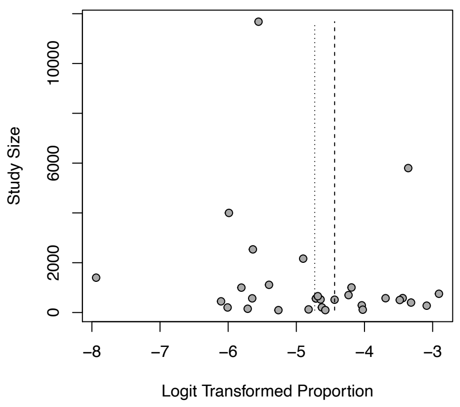

Other findings

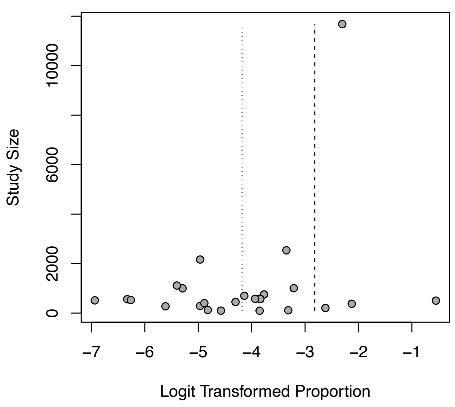

Supplementary Table VI

Regression coefficients for publication year (newer versus older) from multivariable meta-regression models additionally adjusted for age.

**Analysis**	**β (95%CI)**	**p-value**
Vascular		
Aneurysm	-0.000774 (-0.00301 - 0.00147)	0.498
Cavernoma	0.00171 (-0.000445 - 0.00386)	0.120
Other vascular	-0.000431 (-0.0029 - 0.00204)	0.732
Any vascular	3.73e-05 (-0.00323 - 0.00331)	0.982
Neoplastic		
Meningioma	0.00289 (0.000934 - 0.00485)	0.004
Pituitary	0.00166 (0.00023 - 0.00308)	0.023
Glioma	0.000947 (7.06e-05 - 0.00182)	0.034
Other neoplastic	0.00102 (-0.00102 - 0.00307)	0.326
Any neoplastic	0.00416 (0.00206 - 0.00627)	<0.001
Chiari malformation	0.00115 (-0.00192 - 0.00422)	0.464
Pineal cyst	0.00302 (-0.00462 - 0.0107)	0.438
Arachnoid cyst	0.00122 (-0.00194 - 0.00438)	0.450

Supplementary Table VII

Proportions (findings per 1,000 scans) for each analysis with versus without contrast estimated from meta-regression regression models additionally adjusted for age. Proportions relate to the median age for the analysis in question.

**Analysis**	**β (95%CI)**	**Contrast**	**No contrast**	**p-value**
Vascular				
Aneurysm	0.0111 (-0.0224 - 0.0447)	2.87 (0.466 - 6.68)	1.62 (0.086 - 4.34)	0.515
Cavernoma	-0.00663 (-0.0385 - 0.0252)	2.66 (0.409 - 6.24)	3.51 (1.22 - 6.67)	0.683
Other vascular	0.0067 (-0.0258 - 0.0392)	1.08 (0 - 3.73)	0.561 (0 - 2.61)	0.686
Any vascular	-0.0026 (-0.0472 - 0.042)	8.25 (2.82 - 16)	8.76 (3.87 - 15.3)	0.909
Neoplastic				
Meningioma	0.0056 (-0.0298 - 0.041)	6.44 (2.54 - 11.8)	5.5 (2.21 - 9.96)	0.756
Pituitary	0.00537 (-0.019 - 0.0297)	2.45 (0.621 - 5.11)	1.85 (0.528 - 3.73)	0.666
Glioma	-0.00526 (-0.0211 - 0.0105)	0.0484 (0 - 0.645)	0.244 (0 - 0.951)	0.514
Other neoplastic	-0.00105 (-0.0321 - 0.03)	1.82 (0.0913 - 4.9)	1.93 (0.285 - 4.53)	0.947
Any neoplastic	0.00215 (-0.0394 - 0.0437)	13.2 (6.56 - 21.8)	12.7 (7.03 - 19.7)	0.919
Chiari malformation	0.0153 (-0.026 - 0.0567)	4.01 (0.607 - 9.52)	2.06 (0.0606 - 5.8)	0.467
Pineal cyst	-0.0445 (-0.147 - 0.0578)	2.99 (0 - 20.1)	10.7 (1.06 - 28.1)	0.394
Arachnoid cyst	0.0242 (-0.0175 - 0.0658)	11.5 (5.03 - 20.1)	6.63 (2.89 - 11.6)	0.255
